# Molecular subtypes of colorectal cancer in pre-clinical models show differential response to targeted therapies: Treatment implications beyond *KRAS* mutations

**DOI:** 10.1371/journal.pone.0200836

**Published:** 2018-08-17

**Authors:** Rekha Pal, Ning Wei, Nan Song, Shaoyu Wu, Rim S. Kim, Ying Wang, Patrick G. Gavin, Peter C. Lucas, Ashok Srinivasan, Carmen J. Allegra, Samuel A. Jacobs, Soonmyung Paik, John C. Schmitz, Katherine L. Pogue-Geile

**Affiliations:** 1 NSABP/NRG Oncology, Pittsburgh, PA, United States of America; 2 Division of Hematology-Oncology, Department of Medicine, University of Pittsburgh School of Medicine, Pittsburgh, PA, United States of America; 3 Department of Pathology, University of Pittsburgh School of Medicine, Pittsburgh, PA, United States of America; 4 University of Florida Health, Medical Oncology, Gainesville, FL, United States of America; 5 Yonsei University College of Medicine, Seoul, Republic of South Korea; University of South Alabama Mitchell Cancer Institute, UNITED STATES

## Abstract

Molecular subtypes of colorectal tumors are associated with prognosis and prediction for treatment benefit from chemotherapy. The purpose of this study was two-fold: 1) to determine the association of colorectal (CRC) molecular subtypes with response to *targeted* therapies in pre-clinical models and 2) to identify treatments for CRC stem-like subtype because these tumors are associated with a very poor patient prognosis. Eleven CRC cell lines were classified into molecular subtypes and tested for their response to pan-ERBB, MEK, and ERK inhibitors as single agents and in combination. All six inflammatory or TA cell lines were exquisitely sensitive to the combination of MEK and neratinib whereas all five stem-like cell lines were resistant. Growth inhibition in sensitive cell lines was greater with the combination than with either drug alone even in cell lines with *KRAS* mutations. The combination inhibited pERK in inflammatory cell lines but not in four out of five stem-like cell lines. MEK162 plus neratinib were synergistic in cell culture and xenograft models in inflammatory cell lines. The ERK inhibitor, SCH772984, down-regulated pERK, decreased cell viability, and was synergistic with neratinib in both inflammatory and stem-like subtypes. These results suggest that inhibition of pERK is a critical node in decreasing cell viability of stem-like CRC tumors. Our results also suggest that CRC molecular subtypes may yield predictive information and may help to identify patients who may respond to targeted inhibitors.

## Introduction

The current standard of care for stage II/III colon cancer is adjuvant chemotherapy with 5-fluorouracil + leucovorin (FULV) or FULV plus oxaliplatin. The addition of targeted therapies in adjuvant setting has not been shown to reduce recurrences. We examined the association of subtypes with prognosis and for prediction of oxaliplatin benefit, when added to FU/LV by subtyping tumors from patients enrolled into NSABP C-07 (N = 1729), a clinical trial in which patients were randomly assigned to FU/LV with or without oxaliplatin. In agreement with other investigators [1, 2, 3], we showed that patients in C-07 with stem-like/CCS3/CMS4 tumors had a very poor prognosis [4] regardless of whether or not they received oxaliplatin. These data support the clinical utility of molecular subtyping of colon cancer and more importantly, underscores the need to develop new targeted therapies.

Unlike stage II/III disease, the standard of care for colorectal cancer patients with metastatic disease is driven by the presence or absence of *KRAS* mutations. Anti-epidermal growth factor receptor (EGFR) monoclonal antibodies, panitumumab and cetuximab, have been shown to improve overall survival, progression-free survival, and overall response rates in patients with metastatic, *KRAS* WT tumors [5, 6]. However, not all patients with *KRAS* WT tumors respond, and even for those who do, the response is limited [7, 8] by resistance to the anti-EGFR antibodies, which develop within a few months of treatment [9–11].

Preclinical studies showed that resistance to an EGFR blockade consistently displayed persistent activation of mitogen-activated protein kinase (MEK) and extracellular signal-regulated kinase (ERK) irrespective of the upstream genetic alterations [9]. Theoretically, *KRAS* mutants with intrinsic resistance to anti-EGFR antibodies should be sensitive to inhibition of downstream signaling elements. Preclinical models tested this hypothesis with agents targeting pathways downstream of KRAS, however, single-agent inhibitors were disappointing in both PDX models and patients [12–14]. Interestingly, the combination of EGFR and MEK inhibitors was effective in models resistant to anti-EGFR therapies [9]. Recently, Sun and colleagues [15] have shown that a MEK inhibitor as a single agent was ineffective in inhibiting tumor growth in colorectal and lung cancer models, which they observed to be correlated with transcriptional induction of ERBB3. When they combined a MEK inhibitor with a pan-ERBB inhibitor, colorectal cancer cell growth in cell culture and tumor growth *in vivo* were successfully inhibited.

Differences among CRC subtypes with respect to general biology, association with prognosis, and the important clinical observation that oxaliplatin benefit was limited to the enterocyte CRC subtype, led us to hypothesize that subtypes may show a differential response to targeted therapies [4]. To explore whether subtypes would yield predictive information regarding the use of targeted therapies, we used CRC cell lines representing stem-like, transit-amplifying (TA), and inflammatory subtypes with KRAS mutant and WT. These cell lines were tested with pan-ERBB and MEK inhibitors to block upstream and downstream targets of the RAS/RAF pathway, respectively.

## Materials and methods

### Cell lines and subtype classification

All cell lines used in this study were purchased from American Type Culture Collection (ATCC) (Manassas, VA). The CRC cell lines NCI-H747, SW837, SW1116, SW1463, SNU-C1, NCIH508, SW480, SW620, C2BBE1, HS675.T and HCT116 were cultured at 37°C in a humidified atmosphere in the presence of 5% CO_2_. Cells were cultured in DMEM medium with 1X penicillin/streptomycin, and 10% fetal bovine serum. Selumetinib, gefitinib, afatinib, neratinib, MEK162, and SCH772984, were purchased from Selleckchembio (Houston, TX).

### Cell viability assay

CRC cells (8 x 10^3^/well) in 100 μl of growth medium were plated into each 96 microtiter well (Costars: Corning Life Sciences, Cambridge, MA) plates. Cells were allowed to attach for 14 to 18 hours, then were exposed to different doses of inhibitors for 72 hours. The number of viable cells was determined by using the CellTiter-Blue cell viability assay (Promega, WI). The CellTiter-Blue reagent, an indicator dye, was added to cells, incubated for 1 hour, then fluorescence was measured (560(20) _Ex_/ 590(10) _Em_). The release of fluorescence as a result of cellular metabolism indicates living cells. The percent of living cells was calculated by normalizing the absorbance of treated cells to untreated cells. Cells were treated with drug over a range of concentrations. Dimethyl sulfoxide (DMSO) (0.01%) was used as the control treatment. Results are shown as triplicates of mean ± SD.

### PathScan Antibody Array Kit analysis

CRC cells were harvested after 48 hours of culture in the presence of drugs or control vehicle, lysed by using 1X cell lysis buffer containing 1mM PMSF. Cell lysates were subjected to PathScan RTK Signaling Antibody Array Kit (Cell Signaling Technology, Danvers, MA) (Chemiluminescent Readout) as per manufacturer's protocol. Briefly, the PathScan RTK Signaling Antibody Array Kit is a slide-based antibody array founded upon the sandwich immunoassay principle. The array kit allows for the simultaneous detection of 28 receptor tyrosine kinases and 11 important signaling nodes when phosphorylated at tyrosine or other residues. Target-specific capture antibodies using biotinylated protein as positive control, and nonspecific IgG as negative control, were spotted in duplicate onto nitrocellulose-coated glass slides. Cell lysates were then incubated on the slide followed by a biotinylated detection antibody cocktail. Streptavidin-conjugated HRP and LumiGLO reagent were then used to visualize the bound detection antibody by chemiluminescence. An image of the slide was captured with myECL^TM^ Imager (ThermoFisher Scientific, Frederick, MD). Intensities of spots were quantified using myImage analysis software (ThermoFisher Scientific, Frederick, MD).

### Western blot analysis

Protein was extracted from cells using 1X RIPA buffer containing a Halt Protease and Phosphatase Inhibitor Cocktail (ThermoFisher Scientific). Cell lysate proteins (30 μg) were separated on 4–12% NuPAGE Bis-Tris precast gel electrophoresis and transferred to iBlot 2 polyvinyl difluoride membranes (Invitrogen, Carlsbad, CA). The blots were incubated with the appropriate antibodies to detect the protein level of interest, and the immune complexes were visualized by GE Healthcare Amersham ECL WB detection system (ThermoFisher Scientific). Western blots were probed with antibodies against phosphor-ERBB2/HER-2 (Tyr1248) (Cell Signaling Technologies, Danvers, MA; CST# 2247, diluted 1:800), ERBB2 (CST# 2165, diluted 1:800), phosphor-ERBB3 (Tyr1197) (CST# 4561, diluted 1:800), ERBB3 (CST# 4754, diluted 1:800), phosphor-ERK1/2 (CST# 4370, diluted 1:1000), phosphor-p44/p42 MAPK (diluted 1:1000), total p44/42 MAPK, (diluted 1:1000), total ERK1/2 (CST# 9102, diluted 1:1000), phosphor-AKT S473 (CST# 9271, diluted 1:800), AKT (CST# 9272, diluted 1:800), phosphor-EGFR (CST# 3777, diluted 1:800), phosphor-p90RSK (CST# 11989, diluted 1:800), and Actin (Sigma, St. Louis, MO, diluted 1:10,000).

### Mice xenograft model

The animal study protocol was approved by the Institutional Animal Care and Use Committee of the University of Pittsburgh (IACUC protocol #18022278) and in accordance with the National Institutes of Health Guide for the Care and Use of Laboratory Animals. Five million cells per mouse were inoculated subcutaneously in female athymic nude mice (Charles River Laboratories, Wilmington, MA) at the axillary region using human CRC cell lines NCI-H747 (inflammatory subtype) or SW480 (stem-like subtype). Tumor xenografts were allowed to grow to an average size of 100–200 mm^3^ and were randomly assigned to different treatment groups and a vehicle control (0.5% carboxymethylcellulose) (seven mice/group). An initial study was performed for dose tolerability. Efficacy studies were subsequently undertaken with 10 mice in each of 4 cohorts: vehicle, MEK162 alone (3 mg/kg); neratinib alone (10 mg/kg); and MEK162 (3mg/kg) plus neratinib (10mg/kg). Each drug was suspended in 0.5% carboxymethylcellulose and orally administered q.d. Mice were treated daily for 4 weeks. Tumor volume (calculated using the formula: 1/2 (L × W^2^) where L is the longest and W is the shortest axis) and body weight were measured twice weekly. Mice were euthanized by brief isofluorane anesthesia followed by cervical dislocation, two hours following the last dose of drug. Tumors were harvested and snap frozen in a dry ice/ethanol bath for immunoblot analysis. Mice exhibiting any signs of distress including weight loss >20%, tumor size >10% body weight, or tumor growth hindering mouse mobility and ability to eat drink/eat normally, were euthanized.

### Statistical analyses

Five replicate measurements were made for all cellular proliferation and apoptotic assays and these data are presented as a mean plus or minus on standard deviation (SD). Statistical differences were determined by Student *t* test. Results were considered statistically significant with *p* values <0.05. The RTK data was quantitated with a digital imaging system using the myImage Analysis software (ThermoFisher Scientific). Raw data quantification of all figures is shown in S1 File.

## Results

### Selection of colorectal cancer cell lines representing different molecular subtypes

Cell lines were selected for the testing of targeted agents based on *KRAS* mutation status and their molecular subtype. Three of the five CRCA subtypes (inflammatory, TA, and stem-like) were included in this study and represent approximately 80% of stage II and III colon cancer based on our analysis of C-07 [4].

One of the goals of this study was to identify novel treatments for patients resistant to chemotherapy. As noted previously, the stem-like subtype has de novo resistance to chemotherapy and bodes for a poor outcome. Although TA and inflammatory subtypes (approximately 50% of CRC) have a more favorable prognosis when treated in early-stage disease (stage II/III), if disease recurs, these patients have limited options.

**Table 1**shows the mutation status of *BRAF*, *KRAS*, *PIK3CA*, and *NRAS*, as well as the subtype designations of these cell lines based on the results of two other studies [1,16].

**Table 1:** Subtypes and mutation status of colorectal cancer cell lines.

Colon Cancer Cell lines	Sadanandam et al [1]	Medico et al [19]	BRAF	KRAS	PIK3CA	NRAS
C2BBE1	Stem-like	NA	WT	WT	WT	WT
HCT116	Stem-like	Stem-like	WT	Mut	Mut	WT
HS675.T	Stem-like	NA	WT	WT	WT	WT
SW480	Stem-like	Stem-like	WT	Mut	WT	WT
SW620	Stem-like	TA	WT	Mut	WT	WT
NCI-H747	Inflammatory	Inflammatory	WT	Mut	WT	WT
SW837	Inflammatory	Inflammatory	WT	Mut	WT	WT
NCI-H508	TA	TA	Mut	WT	Mut	WT
SNU-C1	TA	TA	WT	WT	WT	WT
SW1116	TA	TA	WT	Mut	WT	WT
SW1463	TA	TA	WT	Mut	WT	WT

Cell line mutations were based on CCLE or Sanger data. Subtypes are based on the articles as indicated. WT: wild type, Mut: mutated.

### Differential effects of RAS/RAF pathway inhibitors by subtype of CRC cell lines

Recently published data has shown combining pan-ERBB and MEK inhibitors demonstrated strong synergistic inhibition of CRC cell proliferation [15]. Therefore, we began our studies by testing MEK inhibitors (selumetinib and MEK162) **(S1A and S1B Fig)**, pan-ERBB inhibitors (afatinib and neratinib) **(S1C and S1D Fig)**, and the EGFR inhibitor, gefitinib, **(S1E Fig)** as single agents in five stem-like cell lines (SW480, SW620, C2BBE1, HS675.T, and HCT116), two inflammatory subtypes (NCI-H747, SW837), and four TA subtypes (SW1116, SW1463, NCI-H508, SNU-C1). The MEK inhibitors (MEK162 and selumetinib) were the most effective agents in decreasing cell viability of inflammatory and TA cell lines **(S1 and S2 Figs)**. The inflammatory cell lines had IC50 values below 1μM, and one TA cell line had an IC50 value at 1μM (MEK162), whereas the other four TA cell lines had IC50 values above 2μM (MEK162), however, IC50 values in stem-like cell lines were all above 2μM, and for a majority of stem-like cell lines, IC50 values were not achieved at concentrations of 5μM (**S1A, S1B, and S2A Figs**). The two inflammatory cell lines, although KRAS mutant, were sensitive to the MEK inhibitors. The pan-ERBB inhibitor, neratinib, had an IC50 above 4μM in both inflammatory, stem-like **(S1D Fig)**, and TA cell lines except NCI-H508 TA cell line, which had an IC50 at 1μM (neratinib) **(S2B Fig).** One TA cell line, NCI-H508, had an IC50 value at 1μM (MEK162); all other TA cell lines had IC50 values from 2μM to 5μM (MEK162) **(S2A Fig)**. Afatinib and gefitinib had little effect on cell viability at concentrations between 125nM to 4μM **(S1C and S1E Figs).**

**Fig 1:**
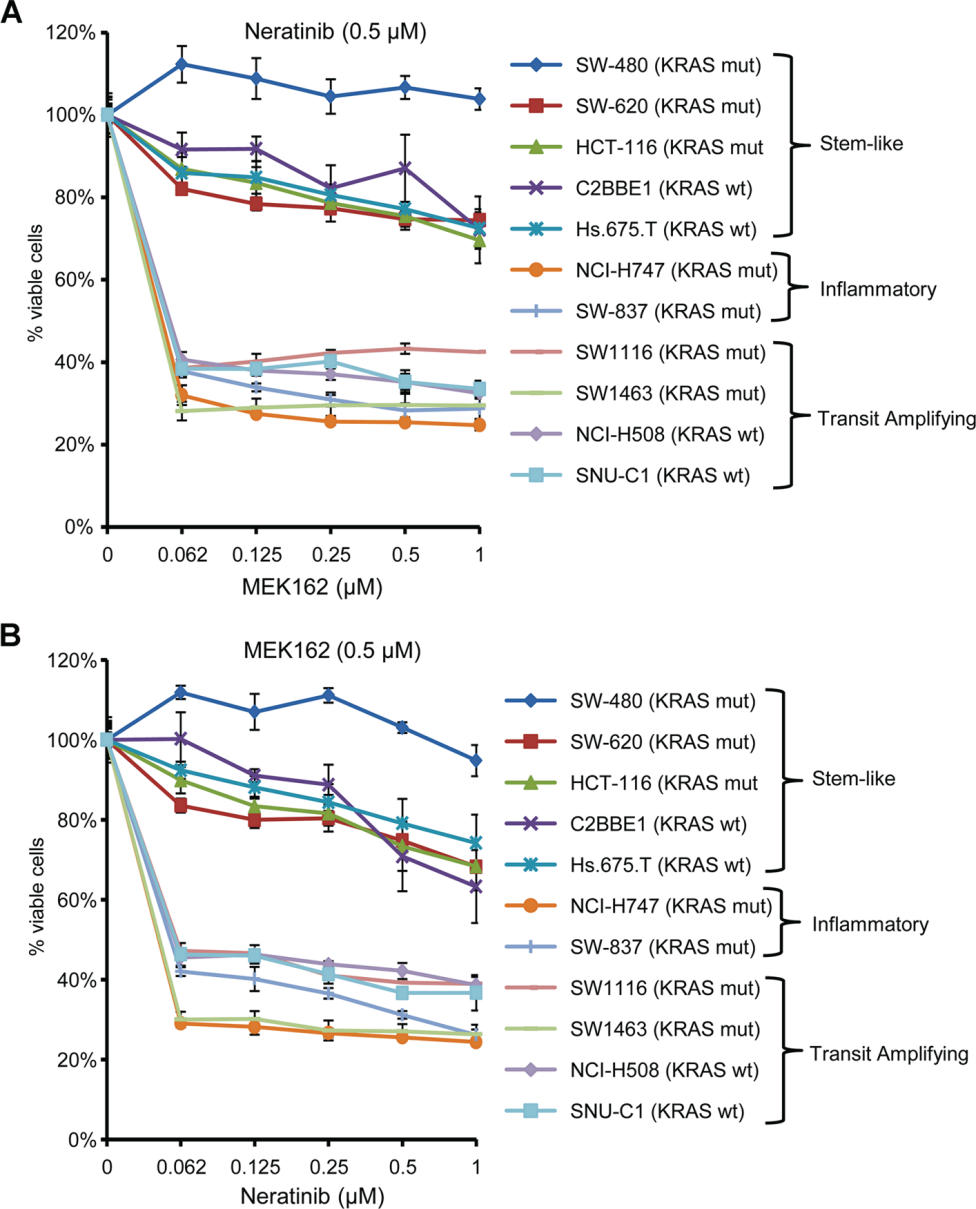
CRC subtype response to MEK162 and neratinib. (**A**) and (**B**): NCI-H747, SW837, SW1116, SW1463, NCI-H508, SNU-C1, SW480, SW620, C2BBE1, Hs675.T, and HCT116 cells were treated with (**A**) a constant dose of neratinib (0.5 μM) in combination with different doses of MEK162, and (**B**) a constant dose of MEK162 (0.5 μM) in combination with different doses of neratinib for 72 hours. Dimethyl sulfoxide (DMSO) (0.01%) was used as the control treatment. Each data point is the mean of five replicates ± SD.

To improve the efficacy of these agents, we combined two different classes of drugs: the MEK inhibitor, MEK162, and the pan-ERBB inhibitor, neratinib. This combination dramatically reduced the cell viability of inflammatory and TA cell lines compared to single-agent treatment **(Fig 1).** For all inflammatory and TA cell lines, the combination of neratinib (0.5 μM) with varying doses of MEK162 (0.062 μM -1μM) treatment was significantly more effective in reducing the cell viability **(Fig 1A)**. The combination of MEK162 at 0.062μM and neratinib at 0.5μM inhibited approximately 70% of inflammatory cell lines and 60% of TA cell lines, but more than 80% of the stem-like cell lines remained viable even at 1μM MEK162 and neratinib at 0.5μM. The resistance of the stem-like cell lines was independent of *KRAS* mutation status. We observed the same pattern of sensitivity and resistance with a constant dose of MEK162 (0.5 μM) with variable doses of neratinib (0.062μM-1 μM) **(Fig 1B).**

We used the combination of neratinib and another MEK inhibitor, trametinib, which has US FDA approval as a single agent for the treatment of patients with BRAF^V600E^-mutated metastatic melanoma. The same pattern of sensitivity was seen when the MEK inhibitor, trametinib, was combined with neratinib **(S3 Fig)**. Stem-like cell lines were resistant to this combination. More than 80% of the stem-like cells remained viable after treatment with 6 nM trametinib and 62 nM neratinib. In contrast, at these same drug concentrations, the viability of inflammatory and TA cell lines ranged between 20–60% **(S3 Fig**). Our data suggest that although intrinsic resistance to EGFR-targeted therapy could be overcome by combination targeted therapies in inflammatory or TA subtypes, this approach would not work for stem-like subtypes.

The synergy between MEK162 and pan-ERBB inhibitors in inflammatory and three out of four TA cell lines with *KRAS* mutations (n = 4) or KRAS wild-type (n = 2) was demonstrated by performing a CompuSyn analysis, which showed a combination index (CI) of ≤1 (CI = 0.08 [NCI-H747] CI = 0.06 [SW837] CI = 0.01 [SW1463] CI = 0.20 [NCI-H508] CI = 0.14 [SNU-C1], indicating that the combination was synergistic **(S4, S5, S6, S7 and S8 Figs).**

### Sensitivity to the combination of MEK162 plus neratinib results in inhibition of ERK phosphorylation (pERK)

**Fig 2:**
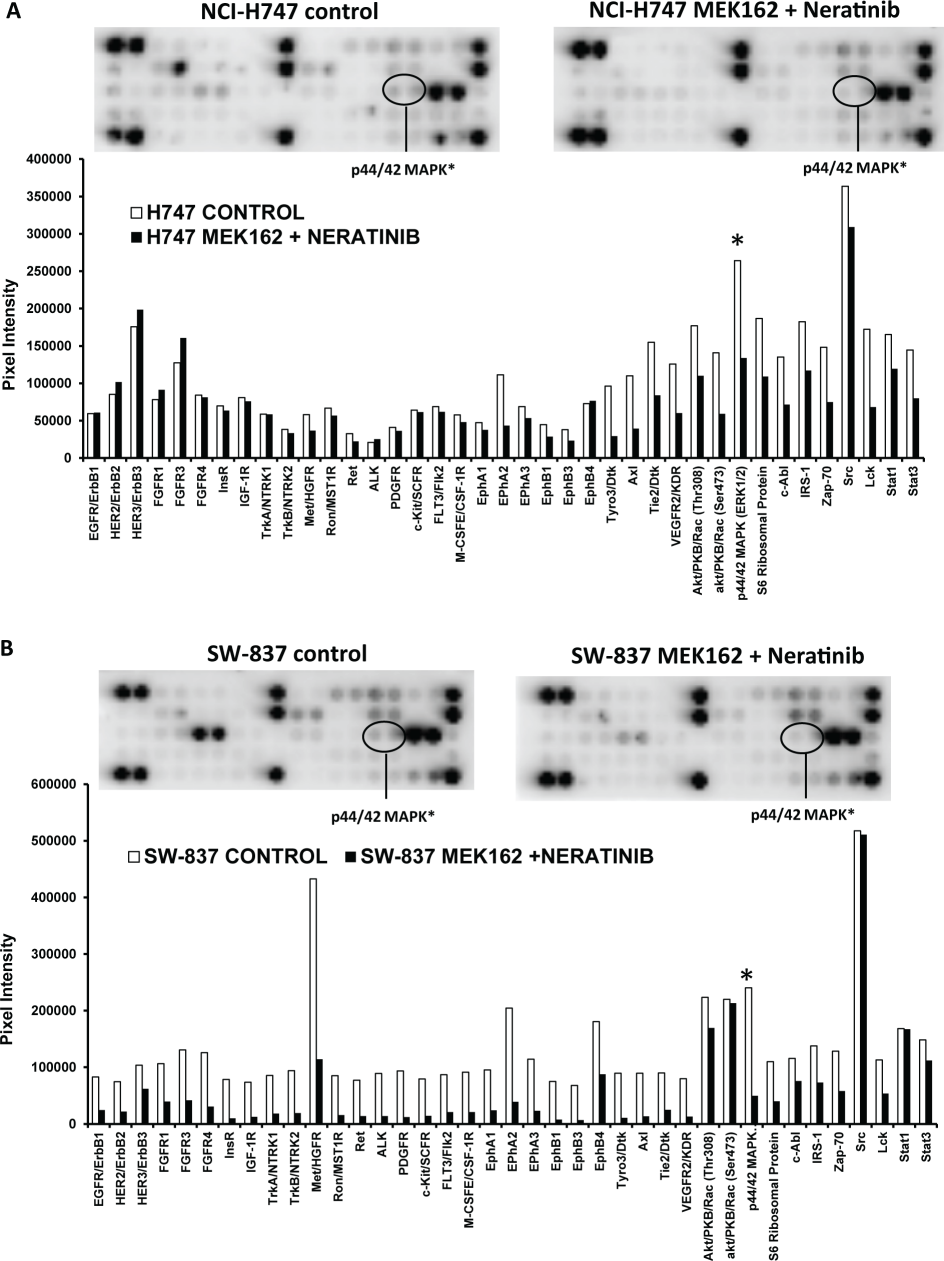
MEK162 plus neratinib inhibits pERK in sensitive (inflammatory) cell lines. Protein profiling of inflammatory cell lines: **(A)** NCI-H747 and **(B)** SW-837, in the absence and presence of MEK162 plus neratinib for 48 hours; protein lysates were prepared and analyzed with the PathScan RTK Signaling Antibody Array Kit. The chemiluminescent film image (upper panel) and the quantification of that image (lower panel) are shown. ERK = extracellular signal-regulated kinase.

**Fig 3:**
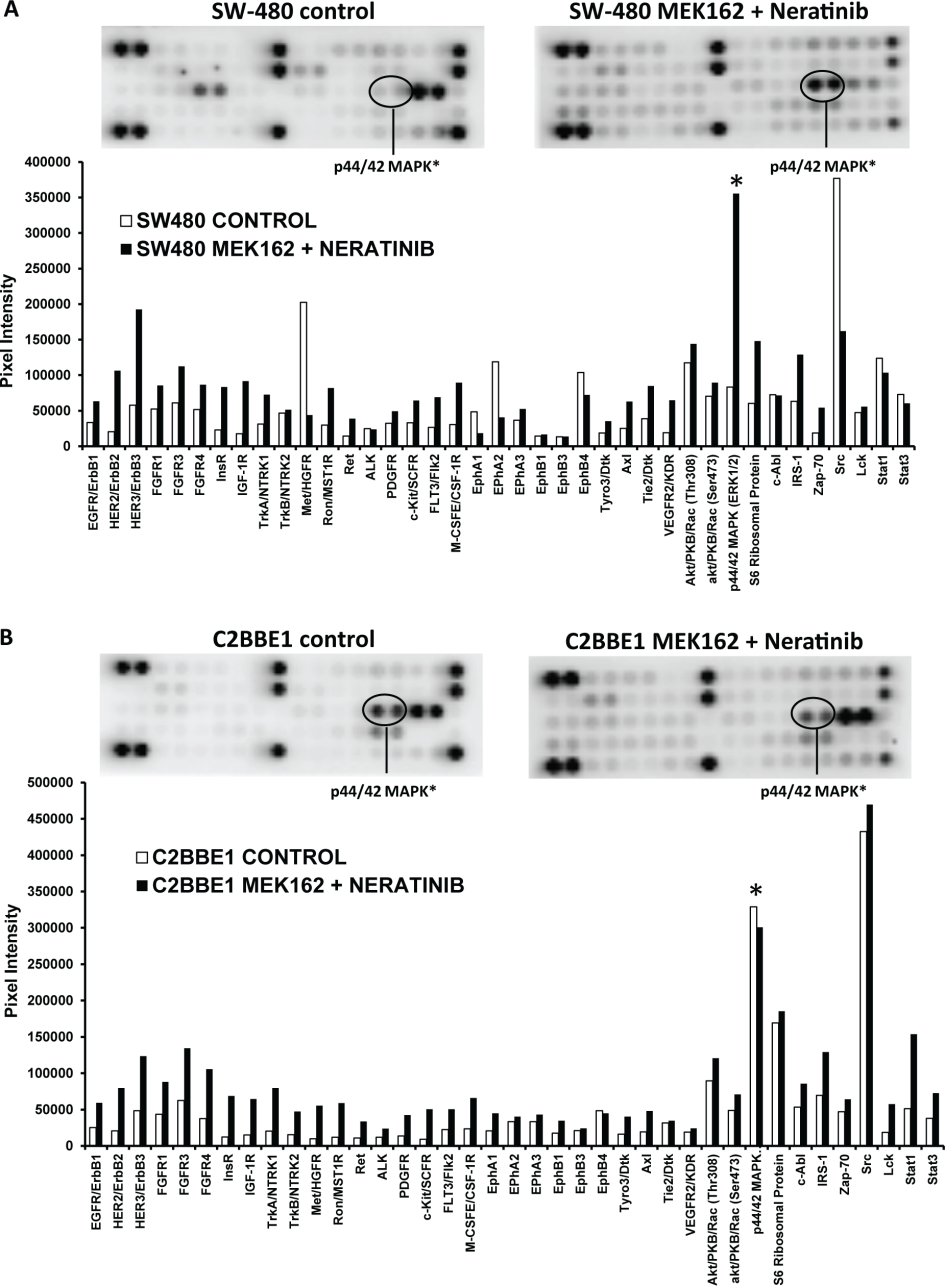
pERK in resistant (stem-like) cell lines after treatment with MEK162 plus neratinib. Protein profiling of stem-like cells lines (A) SW 480 and (B) C2BBE1 were performed as described in **Fig 2**.

To determine the targets that might be responsible for the resistance in cell lines to the combination of MEK162 and neratinib, we used the PathScan RTK Signaling Array Kit. Both inflammatory (n = 2) and stem-like (n = 5) cell lines were incubated with either no drug (control) or with the combination of MEK162 plus neratinib for 48 hours. Thirty-nine different anti-phosph-tyrosine antibodies were interrogated using the PathScan RTK Signaling Antibody arrays. These receptor tyrosine kinases signal primarily through tyrosine phosphorylation events involving a wide range of downstream signaling cascades including the PI3K/Akt, MAPK, and JAK/Stat pathways, which are important for cell division, growth, metabolism, differentiation, migration, and survival. The most dramatic single difference in the phosphorylation levels between the stem-like cell lines (C2BBE1 and SW480) and the inflammatory cell lines (NCI-H747 and SW837), after treatment with the combination of MEK162 plus neratinib, was the phosphorylated protein p44/42 MAPK (also known as pERK1/2). In inflammatory cell lines the levels of pERK1/2 were reduced after treatment with MEK162 plus neratinib **(Fig 2A and 2B)**, however, in the stem-like cell lines the levels of pERK were either increased (SW480) **)** or unchanged (C2BBE1, and HCT-116) **(Fig 3)**. More specifically, inhibition of pERK after treatment with the combination of MEK162 plus neratinib was demonstrated in inflammatory cells lines NCI-H747 (0.50-fold *vs*. control) **(Fig 2A)** and SW837 (0.20-fold *vs*. control) **(Fig 2B)**. Conversely, pERK levels were either increased or unchanged after exposure to the MEK162 plus neratinib combination compared to the control in the stem-like cell lines SW480 (4.2-fold *vs*. control**)**, C2BBE1 (no change *vs*. control**)**, and HCT-116 (1.19-fold *vs*. control **(Fig 3**).

**Fig 4:**
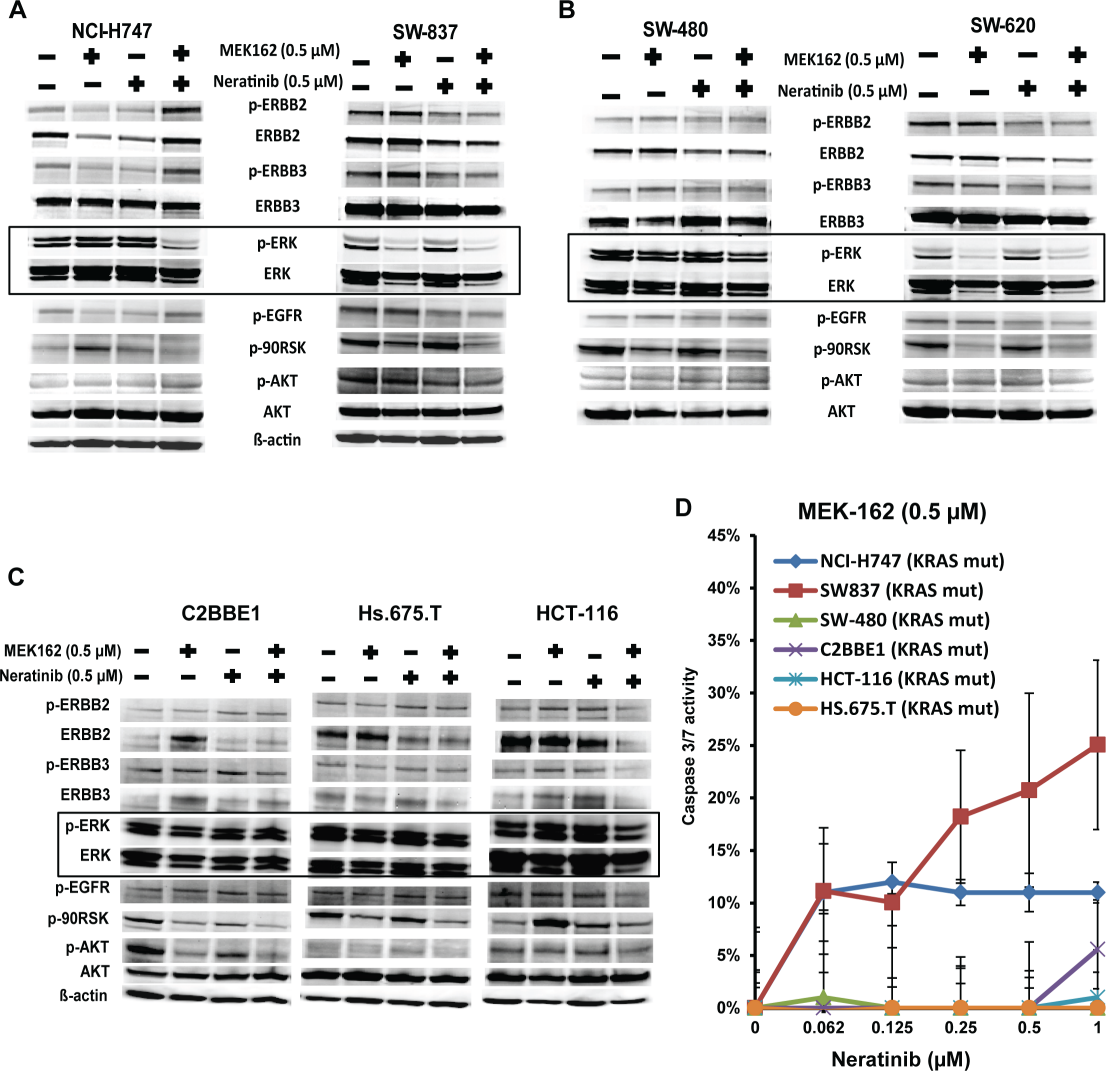
Consistent activation of pERK in stem-like subtype responsible for resistance to the MEK162 plus neratinib combination. **(A)** Effects of MEK162 alone, neratinib alone, and the combination of MEK162 plus neratinib were assessed in sensitive, inflammatory subtype cell lines (NCI-H747, SW-837), **(B)** resistant, stem-like subtype cell lines (SW480, SW620), and **(C)** resistant stem-like subtype cell lines (C2BBE1, Hs.675.T, and HCT-116). Cell lines were cultured with the indicated dosage of MEK162 or neratinib alone, or in combination for 48 hours. Protein expressions were evaluated by western blot analysis with the indicated antibodies. ß-actin was used as a loading control. **(D)** NCI-H747 and SW837 (inflammatory cell lines) and SW-480, C2BBE1, HCT-116, and HS.675.T (stem-like cell lines) were cultured with normal medium as control or medium containing combination of constant dose of 0.5 μM MEK162 with neratinib doses ranging from 0.062 μM to 1 μM. Apoptotic cells were determined by evaluating caspase 3/7 activity using Promega Apo-ONE^TM^ Homogeneous Caspase-3/7 assay. Each data point represents the mean of five replicates; error bars for standard deviation are shown.

To validate the PathScan RTK array results, we examined the RAF-MEK-ERK downstream targets using western blots. Cells were incubated with neratinib or MEK162, singly or in combination for 48 hours. Results were similar to those of the PathScan RTK array. The combination of MEK162 plus neratinib resulted in substantial reduction of pERK levels after 48 hours of treatment in inflammatory cell lines (NCI-H747 and SW837) **(Fig 4A and S2 File)**. Conversely, the MEK162 plus neratinib combination failed to inhibit pERK levels in four out of five stem-like cells lines (SW480 **[Fig 4B andS2 File]**, C2BBE1, Hs.675.T, and HCT-116 **[Fig 4C] S2 File**). Only one stem-like cell line, SW620, showed down-regulation of pERK after exposure to the MEK162 plus neratinib combination **(Fig 4B and S2 File)**. These results suggest that continued activation of pERK might result in resistance in most stem-like cell lines to the combination of MEK162 plus neratinib.

To determine whether apoptosis was responsible for the decreasing cell viability in cell lines when treated with the combination of MEK162 plus neratinib, we assessed apoptosis activity in resistant and sensitive cell lines. Both inflammatory (NCI-H747, SW837) and stem-like (SW-480, C2BBE1, HCT116, HS.675.T) cell lines were treated with the varying doses of neratinib in combination with a constant dose of MEK162 (0.5 μM) for 72 hours followed by evaluation of caspase 3/7 activity. Only the inflammatory cell lines, NCI-H747 and SW837, showed induction of apoptosis after treatment with the combination of MEK162 (0.5 μM) plus neratinib (0.062 μM- 1 μM), whereas stem-like cell lines, SW480,C2BBE1, HCT-116, and Hs.675.T, showed no sign of apoptosis after treatment (**Fig 4D**). This suggests that the decrease of cell viability by the MEK162 plus neratinib combination in inflammatory cell lines may be due to the induction of caspase 3/7 activity, an essential event during apoptosis.

#### MEK162 and neratinib synergistically inhibited tumor growth of inflammatory xenografts but did not inhibit stem-like xenografts

**Fig 5:**
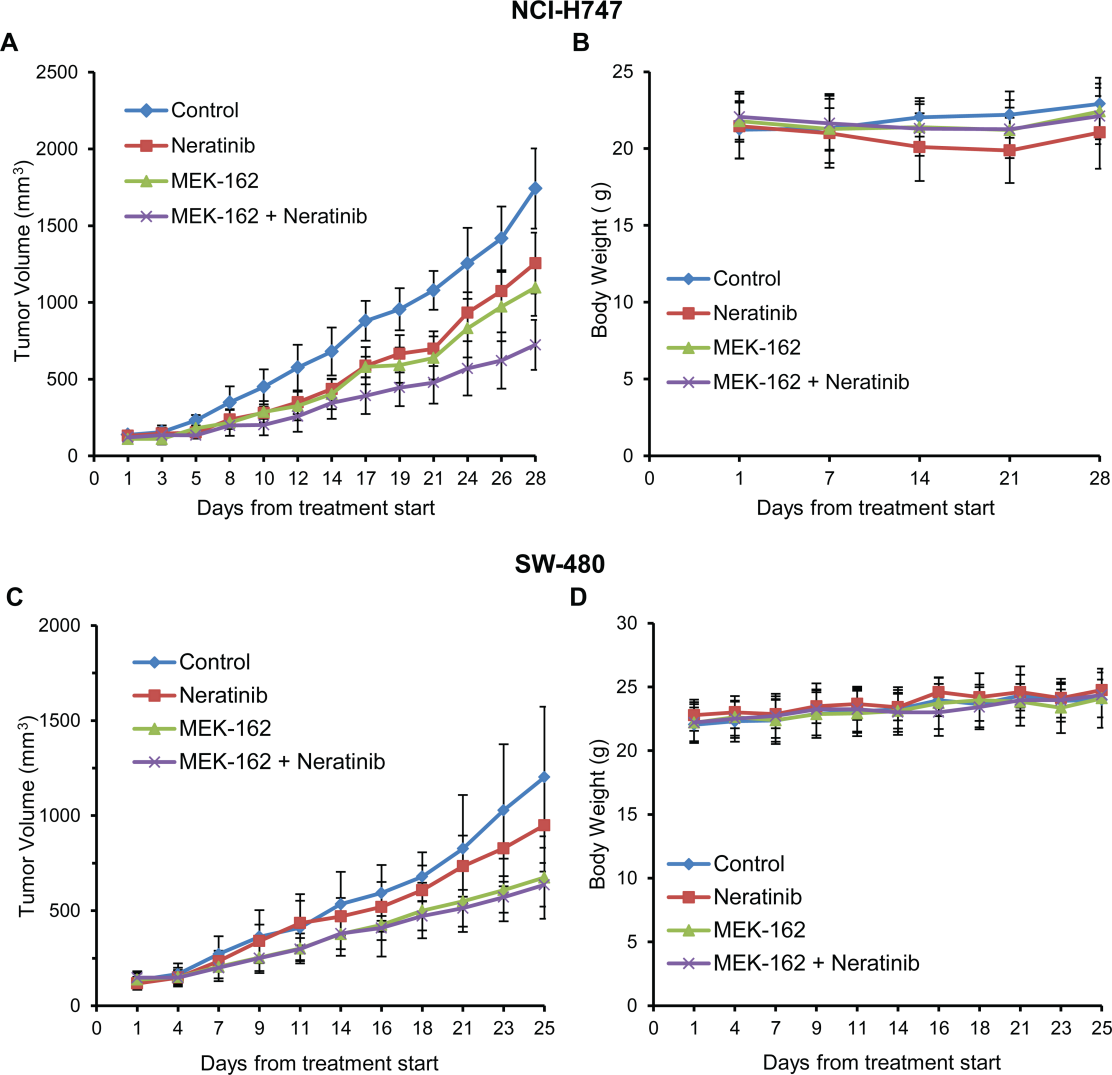
*In-vivo* tumor growth inhibited by combination in inflammatory subtype but not in stem-like. **(A) (B):** NCI-H747 and **(C) (D):** SW480 cells, respectively, were implanted into athymic nude mice. Once xenografts reached ~100 mm^3^, mice were grouped into 4 different cohorts (7 mice/cohort) (control [untreated], neratinib-only (10mg/kg), MEK162-only (3 mg/kg), or MEK162 plus neratinib groups). MEK162 and neratinib were suspended in 0.5% carboxymethyl cellulose and orally administered q.d. for 28 days, respectively. Tumor volumes were measured three times weekly. Volumes represent the mean ± SD of tumor volumes from 7 mice/group. Respective body weights were measured once weekly.

We examined the effect of neratinib and MEK162 as single agents and in combination on an inflammatory (NCI-H747) and a stem-like (SW480) cell line in xenograft models. Both single-agent MEK162 (*p*≤0.002) and single-agent neratinib (*p*≤0.0001) inhibited growth in the inflammatory cell line xenograft. However, the combination resulted in greater tumor inhibition (vehicle control *vs*. combination, *p*≤1.42 x 10^−6^) in the inflammatory cell line than either agent alone **(Fig 5A and Table 2)**. In tumor xenografts, with a stem-like subtype (SW480), MEK162 alone significantly inhibited tumor growth compared to the vehicle control (*p*≤0.006), however, neratinib alone did not (*p* = 0.145) **(Fig 5C)**. Furthermore, the combination of MEK162 plus neratinib had no additive effects on the inhibition of tumor growth compared to the MEK162 alone (*p* = 0.57) in the stem-like xenograft models (**Fig 5C**). No effects on the body weight of the treated xenograft mice were seen (**Fig 5B and 5D**), indicating that there were no toxic effects of treatments. **Table 2**shows the percent of tumor growth inhibition (TGI) in both inflammatory (NCI-H747) and stem-like (SW480) cell line xenograft models in three cohorts: neratinib alone, MEK162 alone, and the combination of MEK162 plus neratinib compared to control.

**Table 2:** Combination of MEK162 plus neratinib inhibits tumor growth in KRAS-mutant inflammatory cell line xenograft.

Tumor line	Tumor type	Subtype status	Mutation status	Dose/Schedule	% TGI* vs control	*p*-value
NCI-H747	colorectal	inflammatory	KRAS mut	Neratinib: 10mg/kg, QD	28%	p≥0.148 vs MEK162
				MEK-162: 3mg/kg, QD	37%	p≥0.148 vs Neratinib
				MEK-162 + Neratinib, QD	58%	p≥1.476E-06 vs Vehicle
				MEK-162 + Neratinib, QD		p≥0.0001 vs Neratinib alone
				MEK-162 + Neratinib, QD		p≥0.002 v MEK162 alone
SW-480	colorectal	stem-like	KRAS mut	Neratinib: 10mg/kg, QD	21%	p≥0.145 vs Vehicle
				MEK-162: 3mg/kg, QD	44%	p≥0.015 vs Neratinib
				MEK-162 + Neratinib, QD	47%	p = 0.5703 vs MEK162

*TGI (tumor growth inhibition)

**Fig 6:**
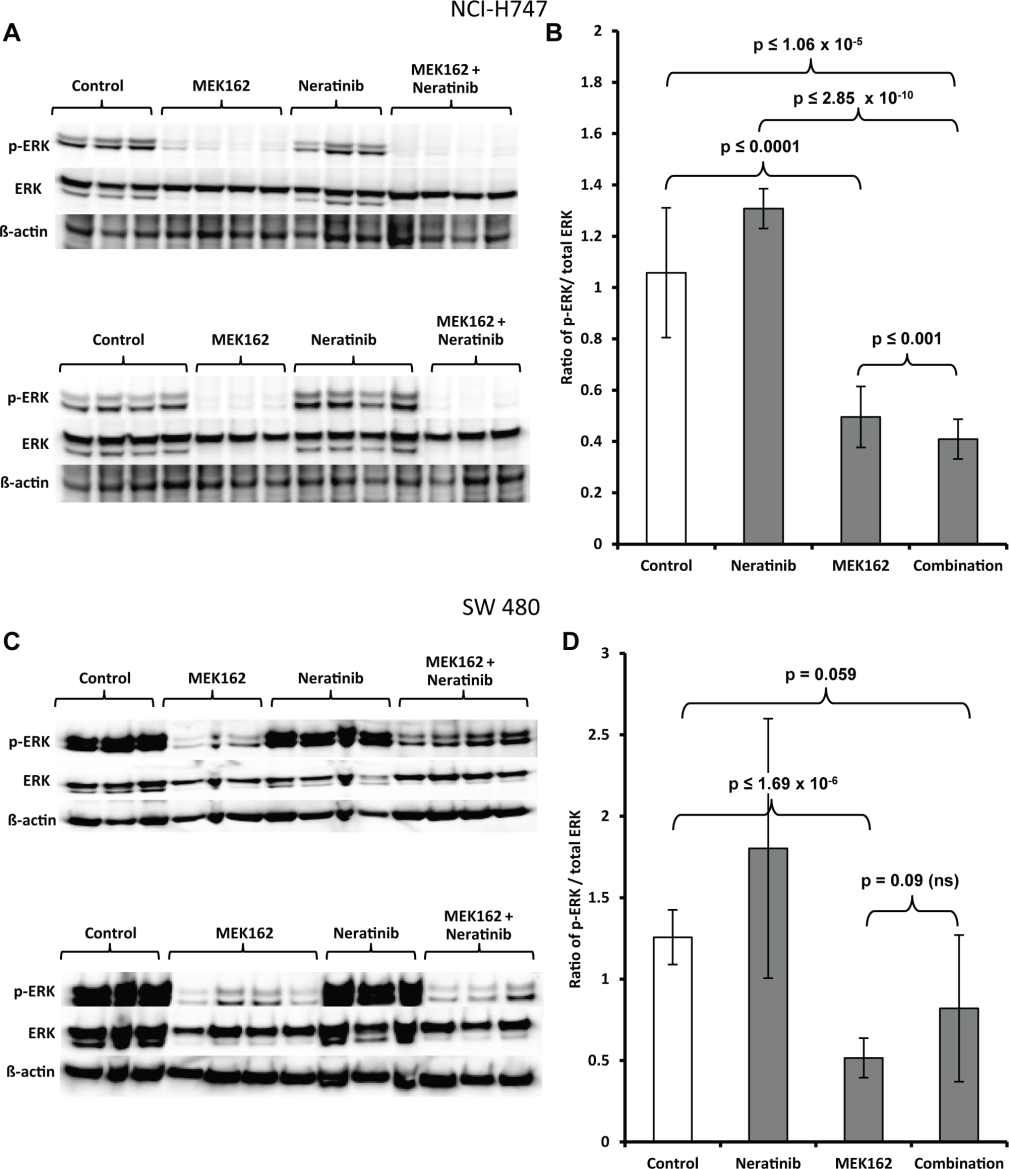
**Western blot analysis of pERK, total ERK, and ß-actin in tumor xenografts of the (A)** immunoblot image, and **(B)** representative digitized values for pERK/total ERK normalized to β-actin from inflammatory subtype cell line NCI-H747, and **(C)** immunoblot image and **(D)** representative digitized values for pERK/total ERK normalized to β-actin from stem-like cell line SW480. Each lane contains a lysate from a single mouse.

Examination of the phosphorylation status from inflammatory and stem-like tumors was carried out on all seven mice from each of four cohorts (control, MEK162 only, neratinib only and combination of neratinib plus MEK162). Single-agent MEK162 significantly reduced the pERK protein levels compared to the control (*p*≤0.0001) in inflammatory subtype tumors **(Fig 6A, 6B and S2 File)**. There was no inhibition of pERK levels by single-agent neratinib. p-ERK levels were significantly downregulated with the combination of neratinib and MEK162 compared to either drug alone (*p*≤0.001 vs. MEK162 only) (p≤2.8511E-10 vs. neratinib only) or control vehicle only (p≤1.060E-05) (**Fig 6A, 6B and S2 File**). In stem-like xenograft models, MEK162 significantly inhibited pERK as a single agent (*p*≤1.696E-06 vs. control), whereas neratinib showed no effect on pERK as a single agent **(Fig 6C, 6D and S2 File)**. pERK levels were not statistically different between MEK162 alone and the combination (*p* = 0.09 vs. combination) in the stem-like xenograft tissue **(Fig 6D and S2 File)**. The inability to inhibit pERK and tumor growth with neratinib alone or in combination with MEK162 was correlated.

### SCH772984, a unique ERK1/2 inhibitor, inhibits cell viability in stem-like subtype cell lines

**Fig 7:**
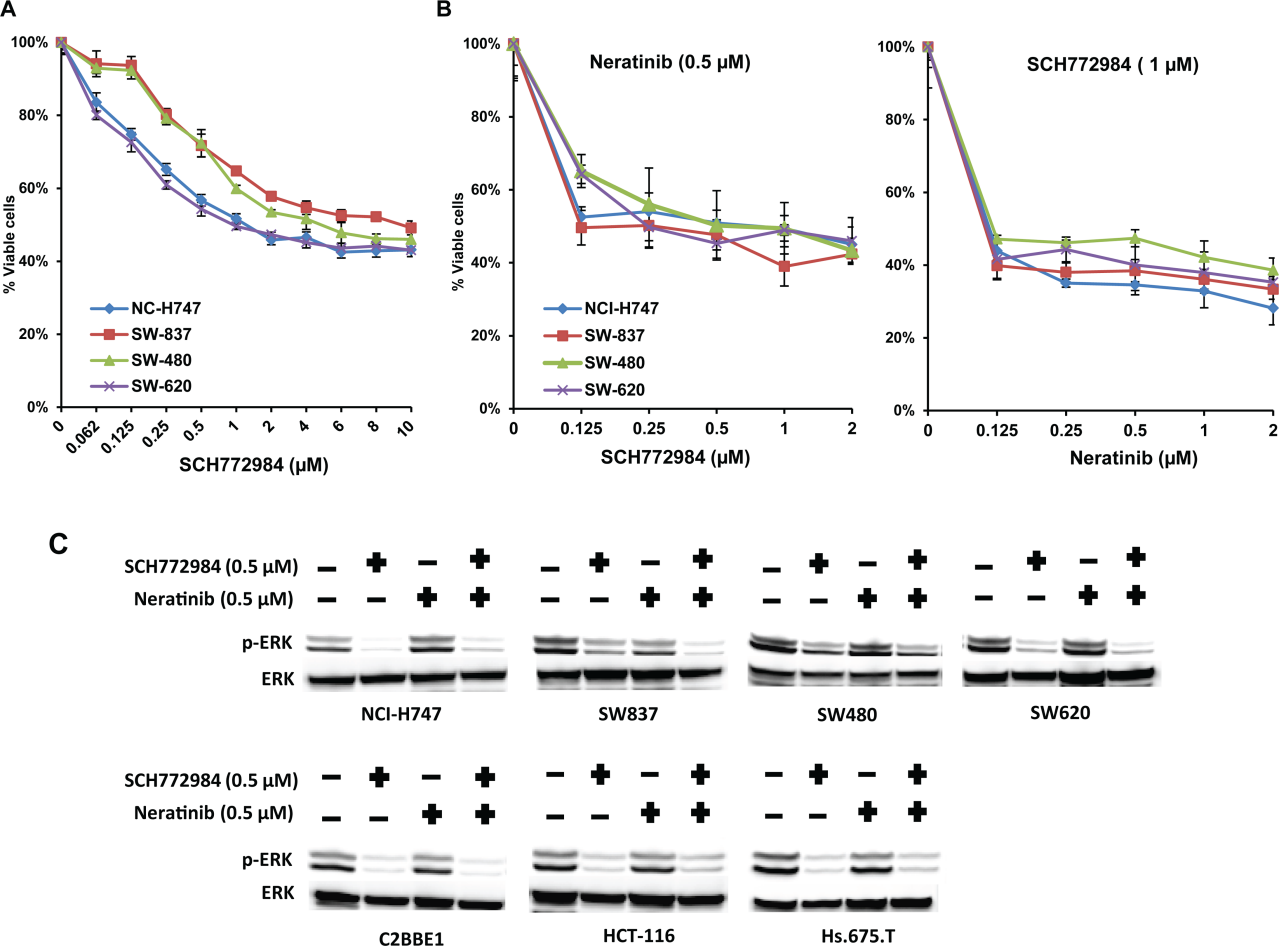
ERK inhibitor SCH772984 inhibits the cell viability of stem-like subtype cell lines. **(A) (B)** NCI-H747, SW837, SW480, and SW620 cells were treated with the ERK inhibitor, **(A)** SCH772984, alone or in combination with **(B)** neratinib at indicated concentrations for 72 hours. Dimethyl sulfoxide (DMSO) (0.01%) was used as the control treatment. Each data point represents the mean of five replicates; error bars indicate one SD. **(C)** Western blot analysis of pERK and total ERK in inflammatory and stem-like cell lines after 48 hours of treatment with SCH772984.

Most of our cell culture and xenograft data show that inhibition of pERK is associated with drug sensitivity. This observation suggests that pERK is a critical downstream target for signaling of the RAS/RAF pathway and led us to test the ERK inhibitor, SCH772984. The SCH772984 is a potent, ATP-competitive and non-competitive inhibitor of ERK1/2 with additional allosteric properties, which inhibits ERK activation/phosphorylation [17, 18]. SCH772984 inhibited cell viability of both inflammatory (IC50 1–2 μM) and stem-like (IC50 1–2 μM) cell lines **(Fig 7A).** The combination of neratinib (0.125 μM) and SCH772984 (1 μM) was more effective at decreasing cell viability in both inflammatory and stem-like cell lines than either drug alone **(Fig 7B).** Moreover, inhibition of pERK was seen with single-agent SCH772984 in both inflammatory and stem-like cell lines and correlated with loss of cell viability **(Fig 7C)**. CompuSyn analysis showed that the combination of SCH772984 plus neratinib were highly synergistic CI ~0.19) in both inflammatory and stem-like subtypes (**S9, S10, S11, and S12 Figs**).

## Discussion

In this study, we demonstrated that the combination of MEK162 plus neratinib effectively inhibited cell viability in inflammatory or TA subtypes but not in the stem-like subtype. Tumor growth of an inflammatory cell line in mouse-xenograft models was inhibited by MEK162 and neratinib as single agents, however, the combination of MEK162 plus neratinib was more efficacious, synergistic, and independent of KRAS status. The finding that sensitivity appeared independent of KRAS status is a particularly important observation as currently there are no specific therapies for KRAS MT tumors [4]. In contrast, the stem-like xenograft model did not respond to the combination of MEK162 plus neratinib.

Sun et al. [15], who tested other MEK and pan-ERBB inhibitors, showed that combining MEK and pan-ERBB inhibitors overcame the intrinsic resistance to MEK inhibition alone. They postulated that resistance to MEK inhibition was the result of upregulation of ERBB3, which was overcome by combining MEK and pan-ERBB inhibitors. In contrast, although we showed that combining MEK162 with the pan-ERBB inhibitor, neratinib, was effective in decreasing the cell viability of both inflammatory cell lines, NCI-H747 and SW837, we were unable to demonstrate any change in pERBB3 expression with MEK inhibition alone or in combination (**Fig 4A and S2 File**). Moreover, in resistant stem-like cell lines, we did not observe any change in ERBB3 expression levels with MEK, neratinib, or the combination (**Fig 4B** and **4C, S2 File**). One plausible explanation for the difference in our findings and those of Sun et al., is that we used different pan-ERBB and MEK inhibitors, which may affect the amount of ERBB-RAS-RAF-ERK targets downregulation and assessment.

Other investigators [19, 20] have shown a direct correlation between inhibition of pERK and sensitivity of targeted agents. In the inflammatory xenograft, MEK162 alone, but not neratinib, inhibited pERK. However, inhibition of pERK was statistically significant with the combination of MEK plus neratinib compared to MEK alone (p≤0.001 vs. MEK162 only). In contrast, in a stem-like xenograft model, there was no additive effect of neratinib with MEK162 on pERK levels. This suggests that downregulation of pERK correlated with the sensitivity to the combination in the inflammatory subtype. On the other hand, consistent activation of pERK may be a resistant marker in the stem-like subtype. Targeted therapies such as neratinib and MEK162, if effective, should inhibit critical kinases in the RAF-MEK-ERK pathway. Persistent ERK activation may explain resistance to the MEK162 and neratinib combination in stem-like tumors. In an attempt to downregulate pERK, we tested SCH772984, a potent and selective ERK inhibitor targeting MAPK signaling and demonstrated to inhibit cell proliferation in BRAF- or MEK resistant model [17]. SCH772984 significantly down-regulated pERK levels with inhibition in cell viability in stem-like cell lines resistant to MEK plus neratinib.

Patients with stem-like tumors represent approximately 25% to 30% of the CRC population and have a very poor prognosis regardless of treatment [4] and thus are in need of new therapeutic options. We observed that the ERK inhibitor, SCH772984, was able to decrease the cell viability of stem-like cell lines and that the combination of SCH772984 and neratinib was highly synergistic in inhibiting the cell viability of both inflammatory and stem-like cell lines. The inhibition of cell viability correlated with the inhibition of pERK. The combination of an ERK inhibitor and pan-ERBB inhibitor may represent a therapeutic approach, which should be further tested in patients with stem-like CRC tumors.

Molecular subtyping of colorectal cancer with gene expression represents a relatively new approach to predicting treatment response. The development of a validated assay may be a valuable tool not only in determining prognosis but also by guiding more precise therapies.

## Supporting information

S1 Fig Viability assays of inflammatory and stem-like cell lines after exposure to EGFR, ERBB2, and MEK inhibitors. (TIF)

S2 Fig Cell line viability assays of TA cell lines after exposure to ERBB2 and MEK inhibitors. (TIF)

S3 Fig Cell line viability assays of inflammatory, stem-like, and TA cell lines after exposure to trametinib and neratinib. (TIF)

S4 Fig CompuSyn analysis of cell line, NCI-H747, after exposure to MEK162 plus neratinib. (TIF)

S5 Fig CompuSyn analysis of cell line, SW837, after exposure to MEK162 plus neratinib. (TIF)

S6 Fig CompuSyn analysis of cell line, SW1463, after exposure to MEK162 plus neratinib. (TIF)

S7 Fig CompuSyn analysis of cell line, NCI-H508, after exposure to MEK162 plus neratinib. (TIF)

S8 Fig CompuSyn analysis of cell line, SNU-C1, after exposure to MEK162 plus neratinib. (TIF)

S9 Fig CompuSyn analysis of cell line, NCI-H747, after exposure to SCH772984 and neratinib. (TIF)

S10 Fig CompuSyn analysis of cell line, SW837, after exposure to SCH772984 and neratinib. (TIF)

S11 Fig CompuSyn analysis of cell line, SW480, after exposure to SCH772984 and neratinib. (TIF)

S12 Fig CompuSyn analysis of cell line, SW620, after exposure to SCH772984 and neratinib. (TIF)

S1 File Raw data quantification. (XLSX)

S2 File Uncropped western blots / Raw data. (PDF)

## Abbreviations

ATCC: American Type Culture Collection
CI: combination index
CRC: Colorectal Cancer
CRCSC: Colorectal Subtyping Consortium
CMS: Consensus molecular subtypes
DMSO: Dimethyl sulfoxide
EGFR: Epidermal growth factor receptorp
ERK: Extracellular signal-regulated kinase
FOLFIRI: fluorouracil, leucovorin, and irinotecan
MEK: Mitogen-activated protein kinase
pERK: phosphorylated ERK
TA: Transit amplifying
WT: Wildtype
