# Supplementary figures and images for: Molecular subtypes of colorectal cancer in pre-clinical models show differential response to targeted therapies: Treatment implications beyond KRAS mutations
Source: PLoS One. 2018 Aug 17;13(8):e0200836. doi: 10.1371/journal.pone.0200836 (PMC6097647; doi:10.1371/journal.pone.0200836)

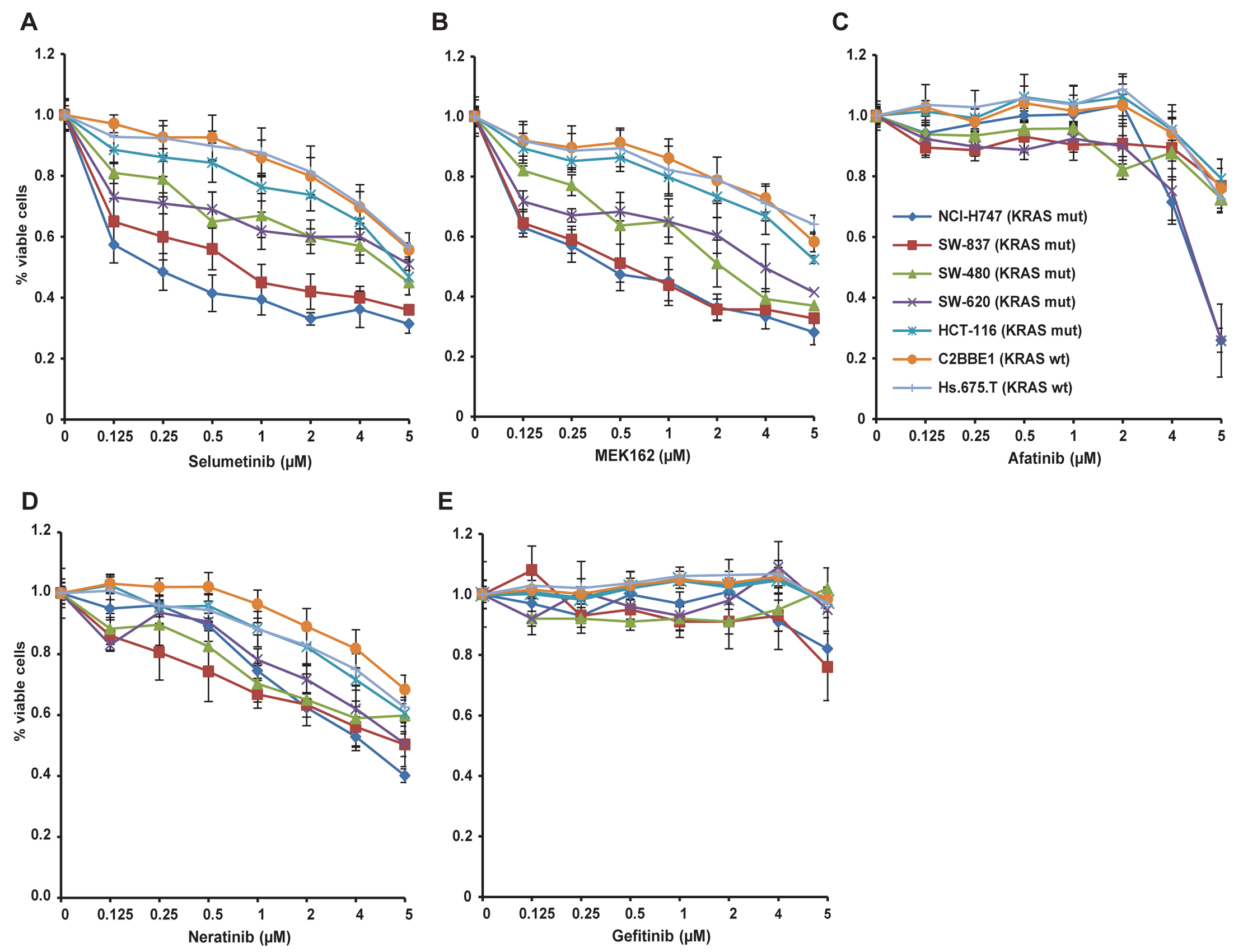

Supplement: S1 Fig — (TIF) [file pone.0200836.s001.tif]

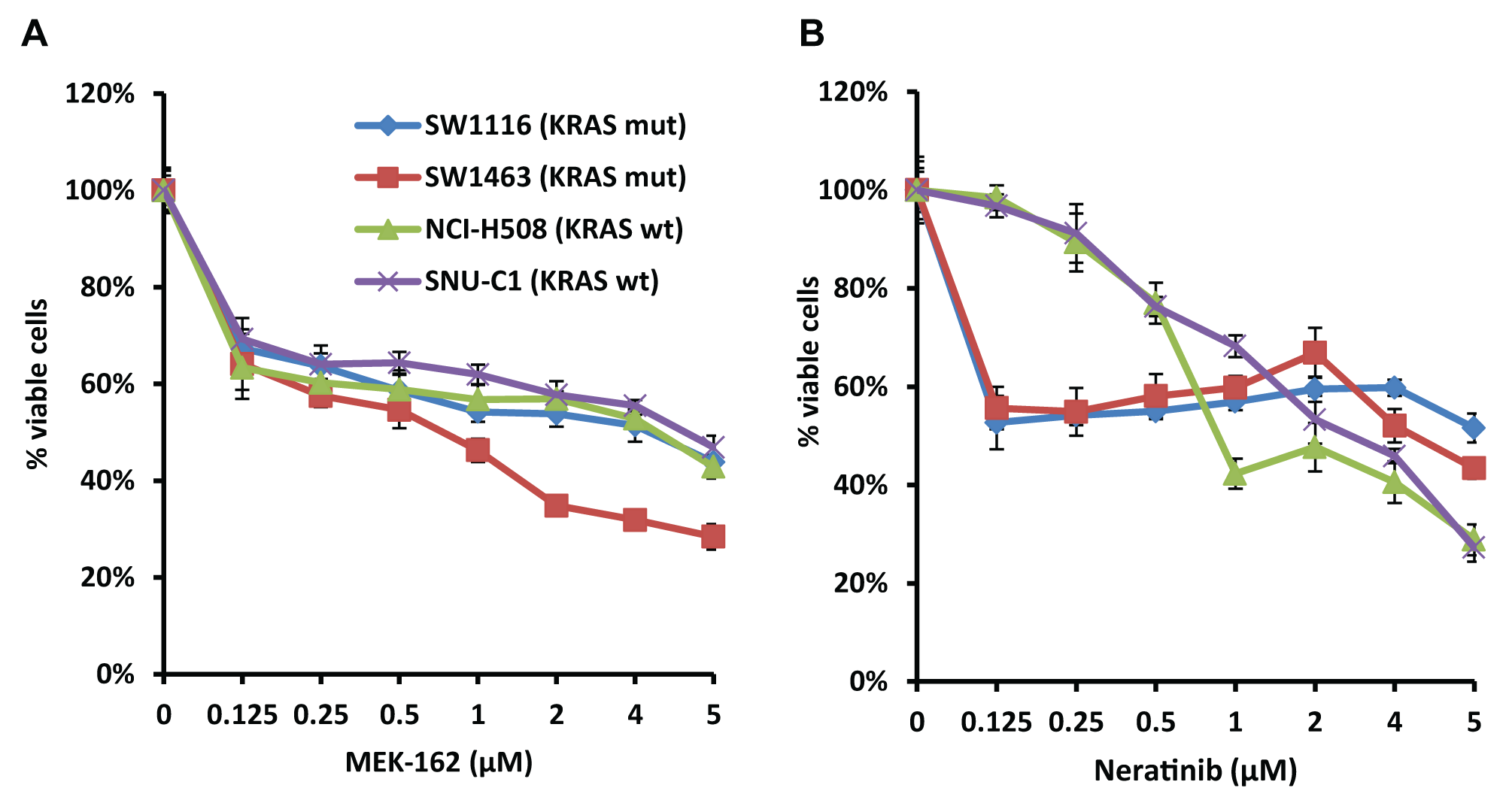

Supplement: S2 Fig — (TIF) [file pone.0200836.s002.tif]

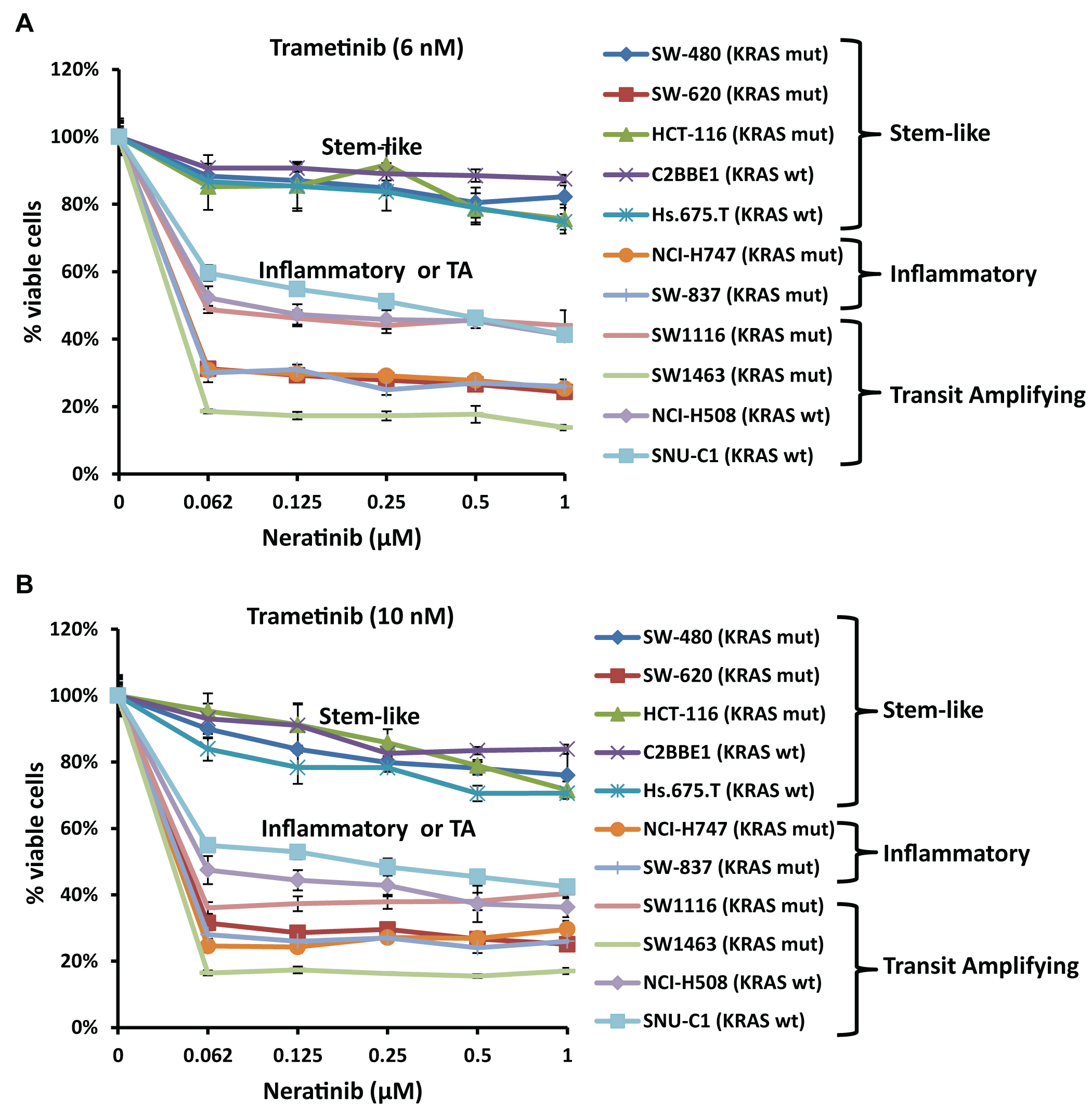

Supplement: S3 Fig — (TIF) [file pone.0200836.s003.tif]

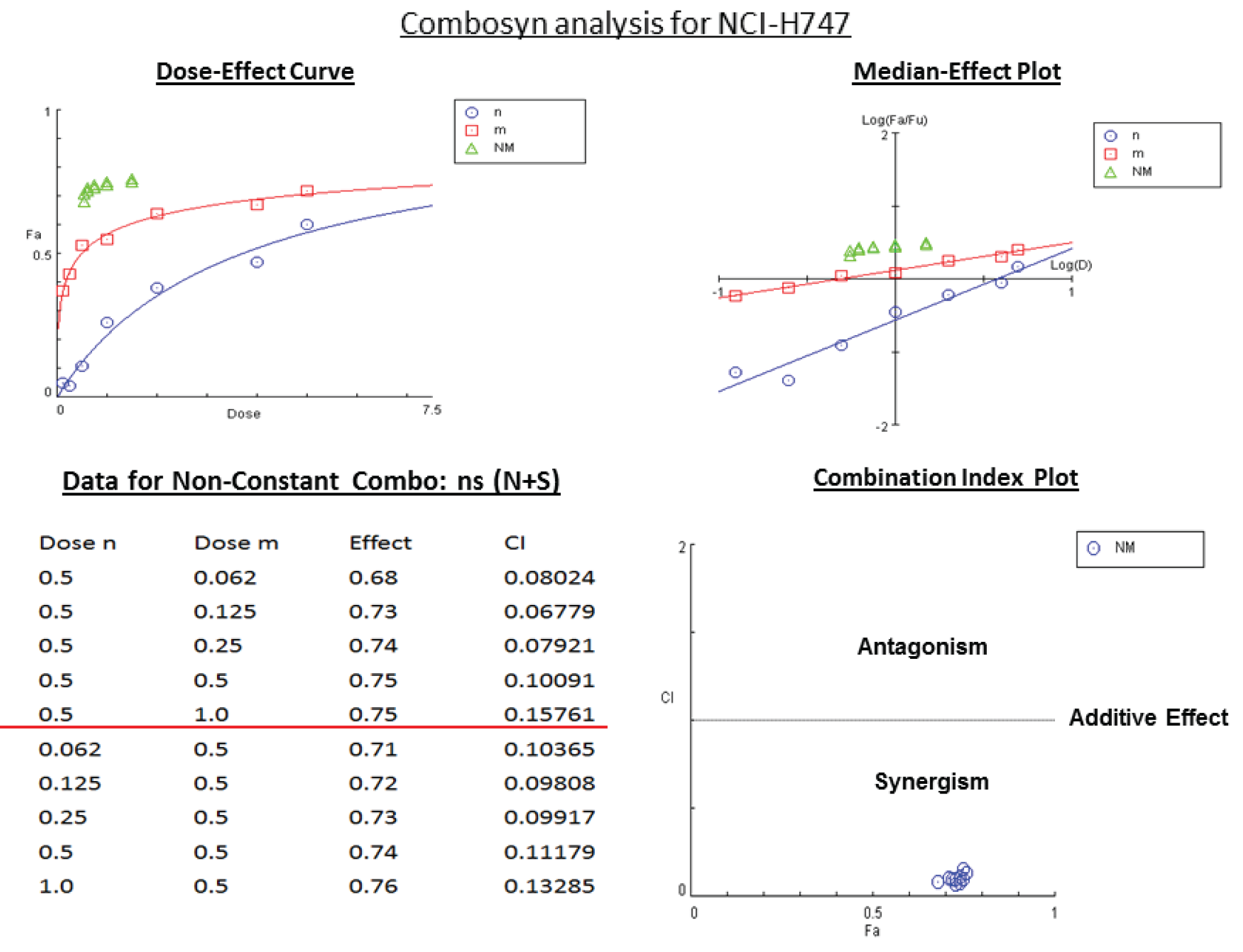

Supplement: S4 Fig — (TIF) [file pone.0200836.s004.tif]

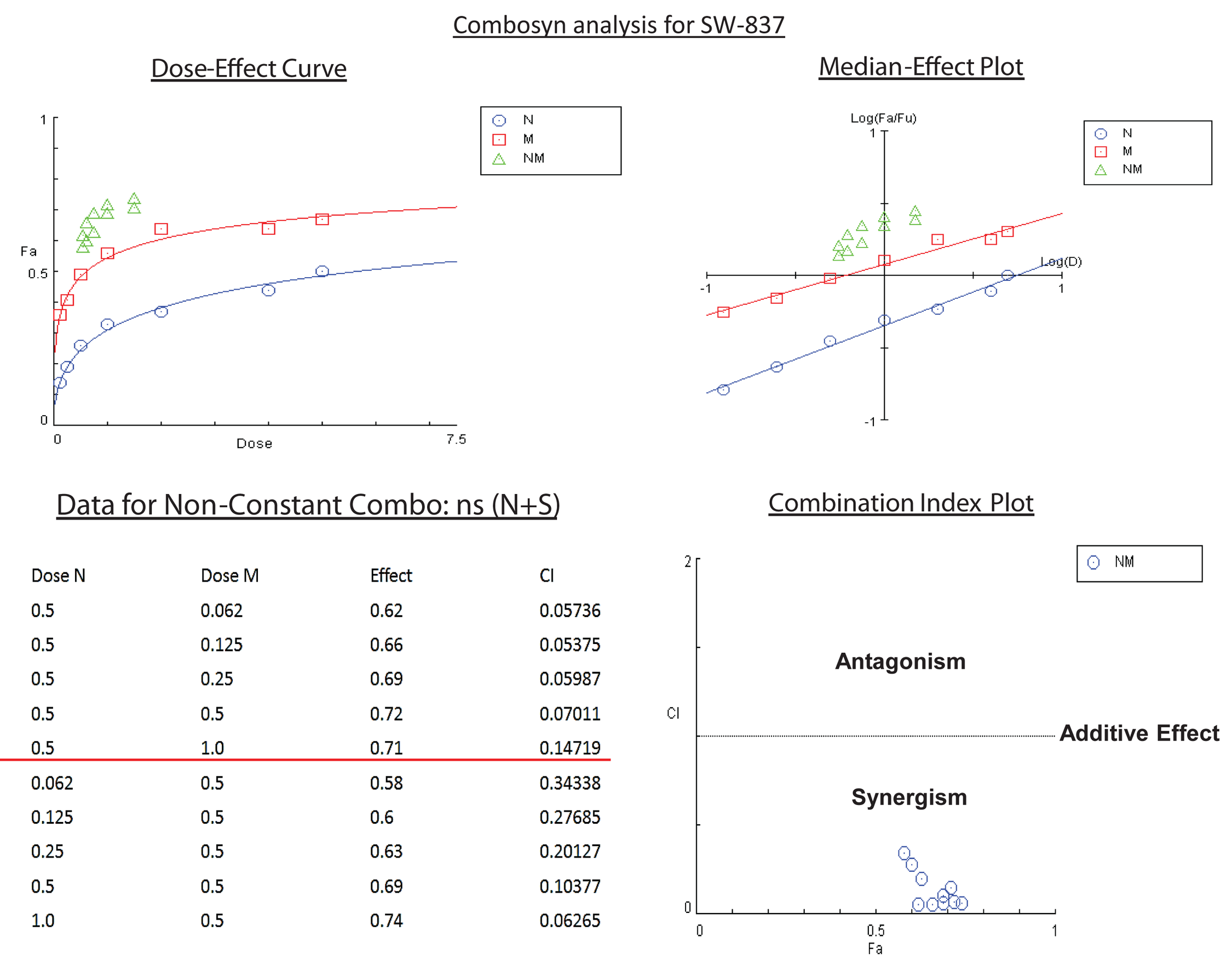

Supplement: S5 Fig — (TIF) [file pone.0200836.s005.tif]

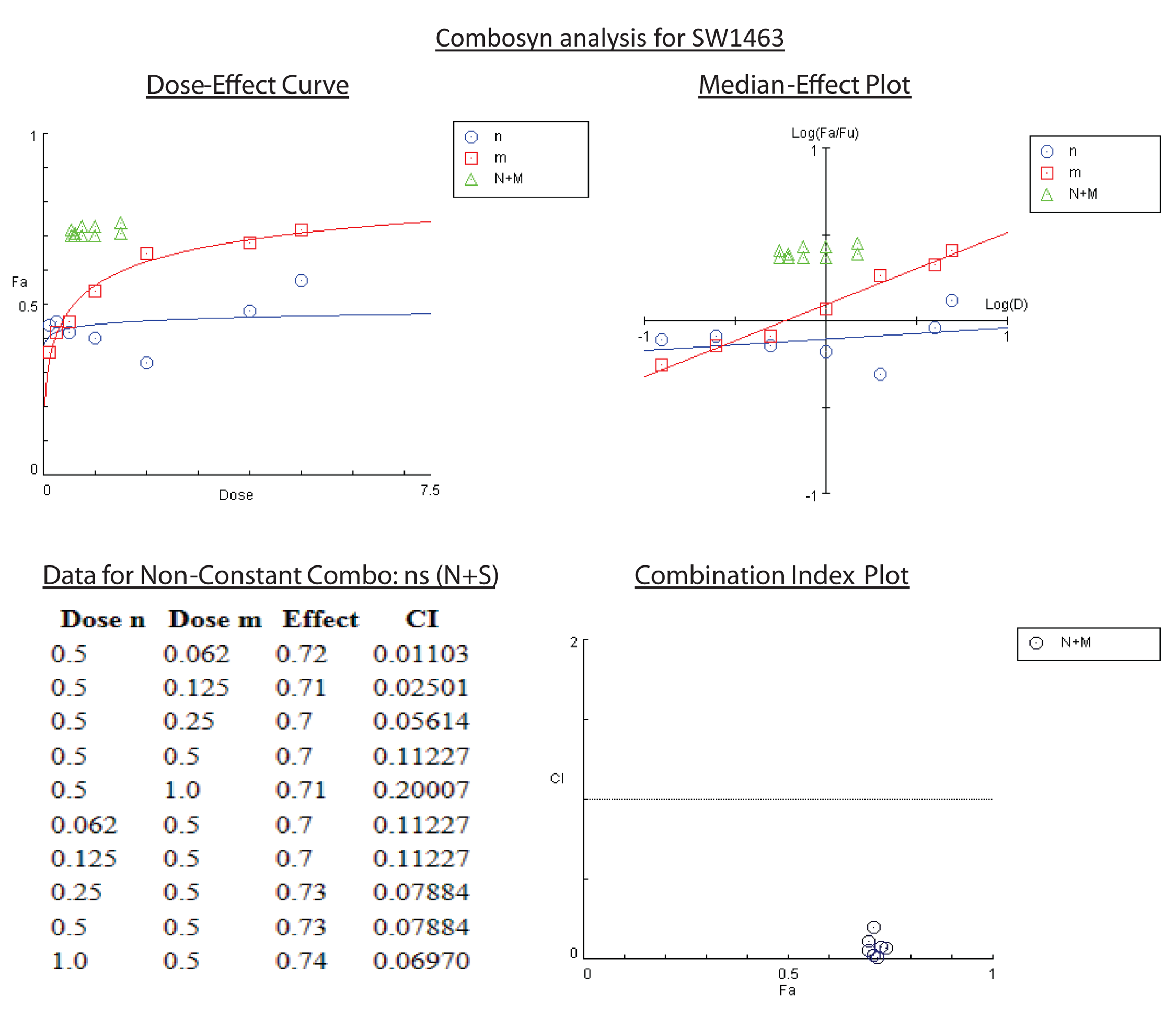

Supplement: S6 Fig — (TIF) [file pone.0200836.s006.tif]

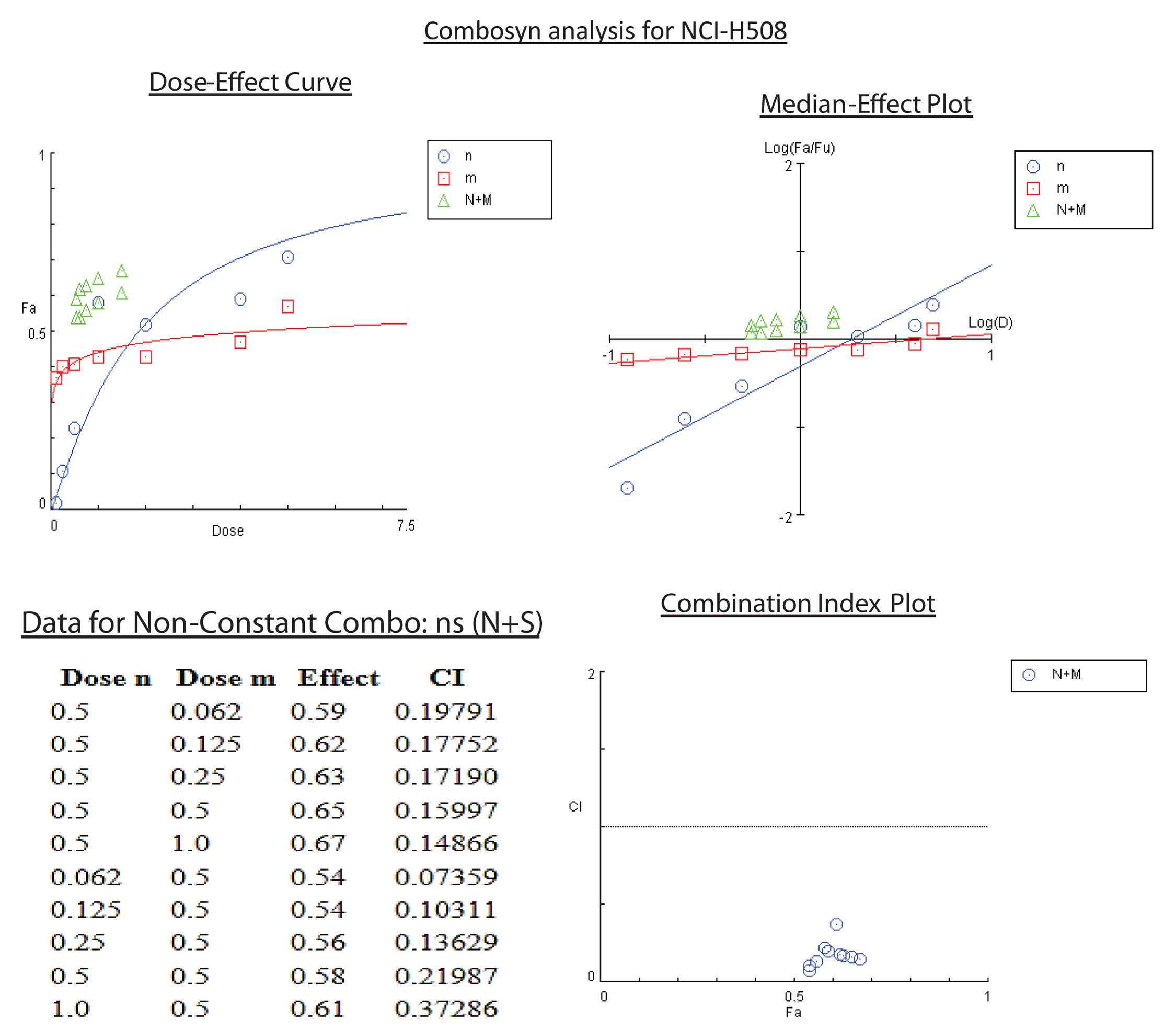

Supplement: S7 Fig — (TIF) [file pone.0200836.s007.tif]

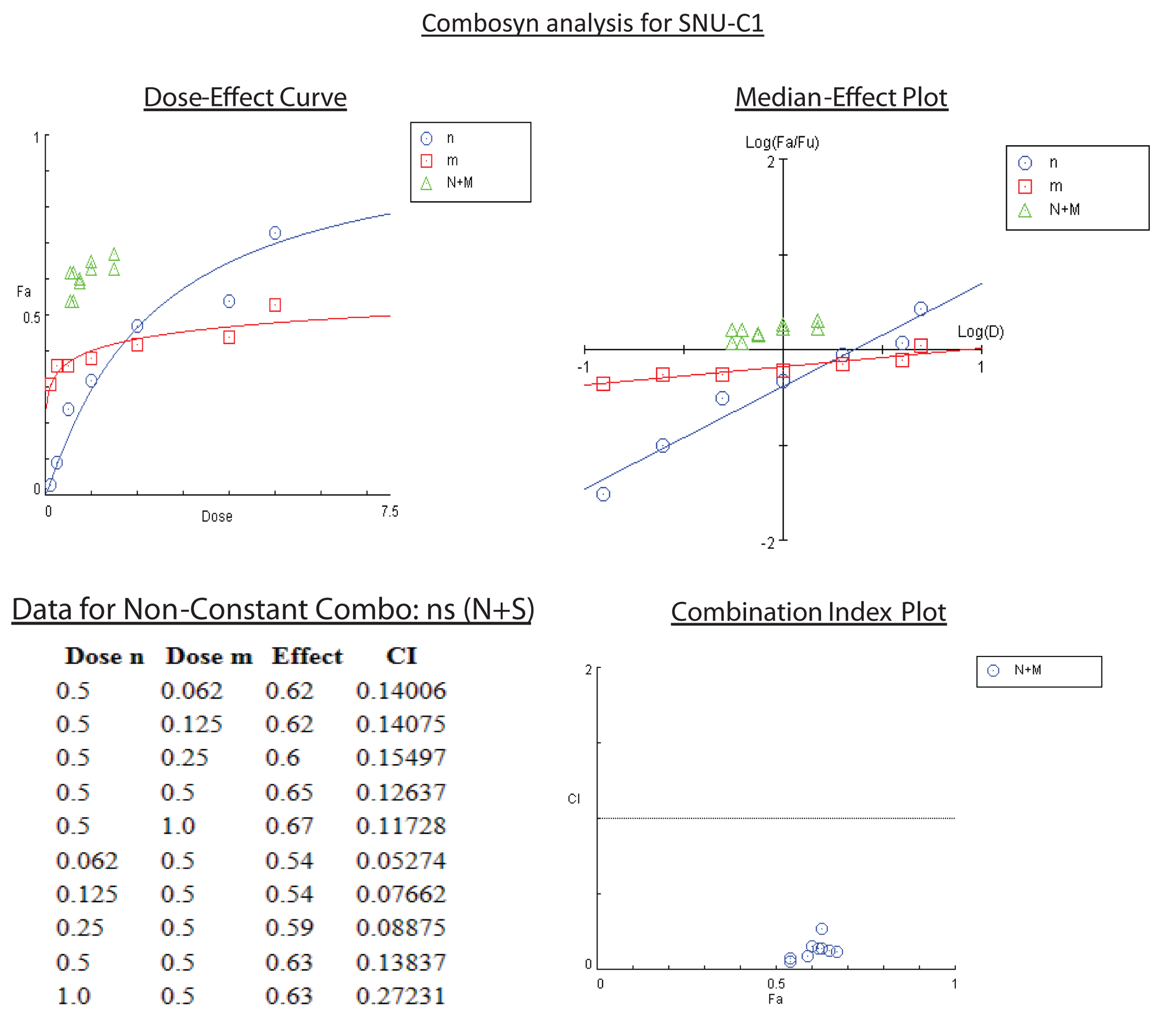

Supplement: S8 Fig — (TIF) [file pone.0200836.s008.tif]

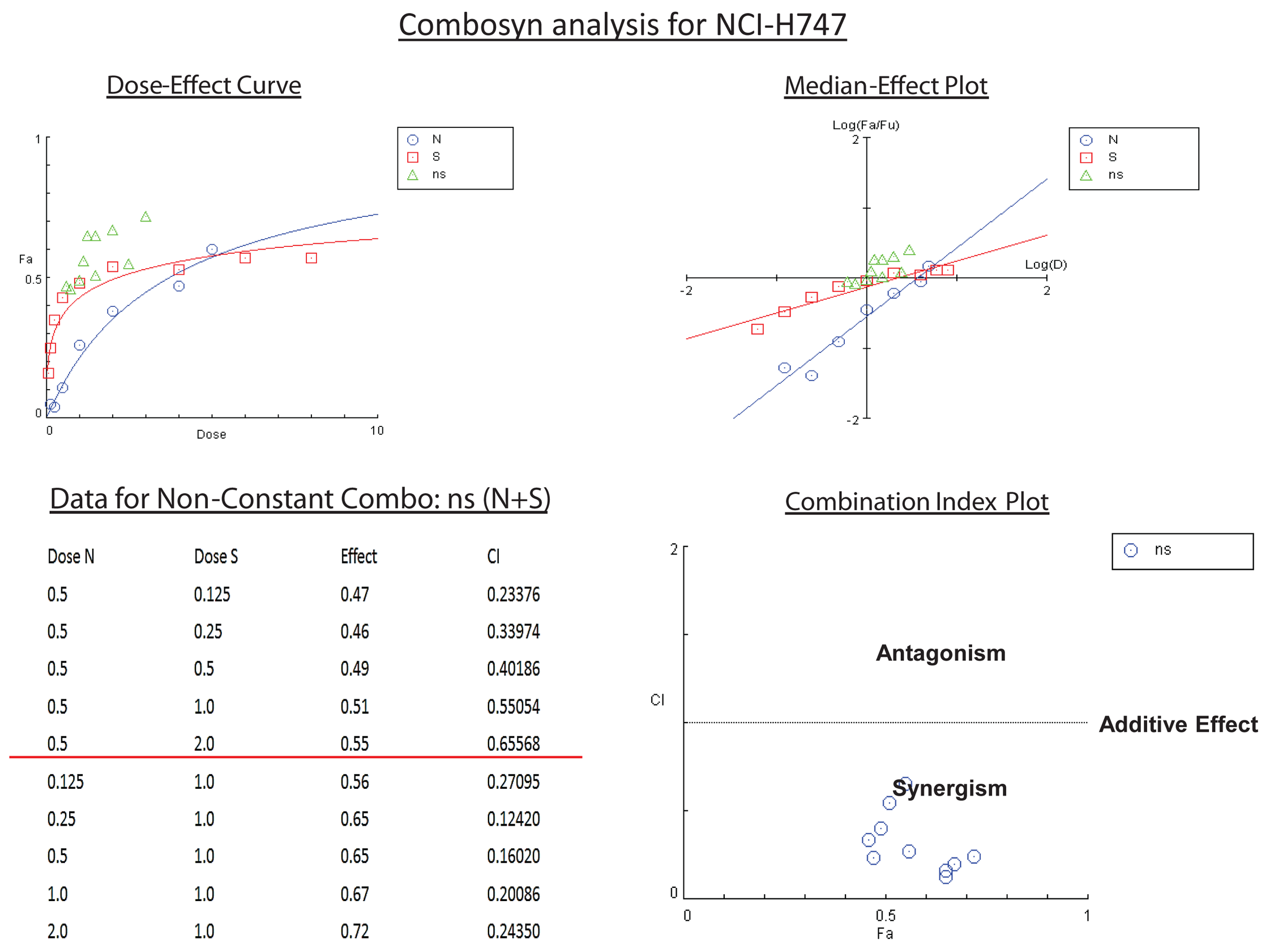

Supplement: S9 Fig — (TIF) [file pone.0200836.s009.tif]

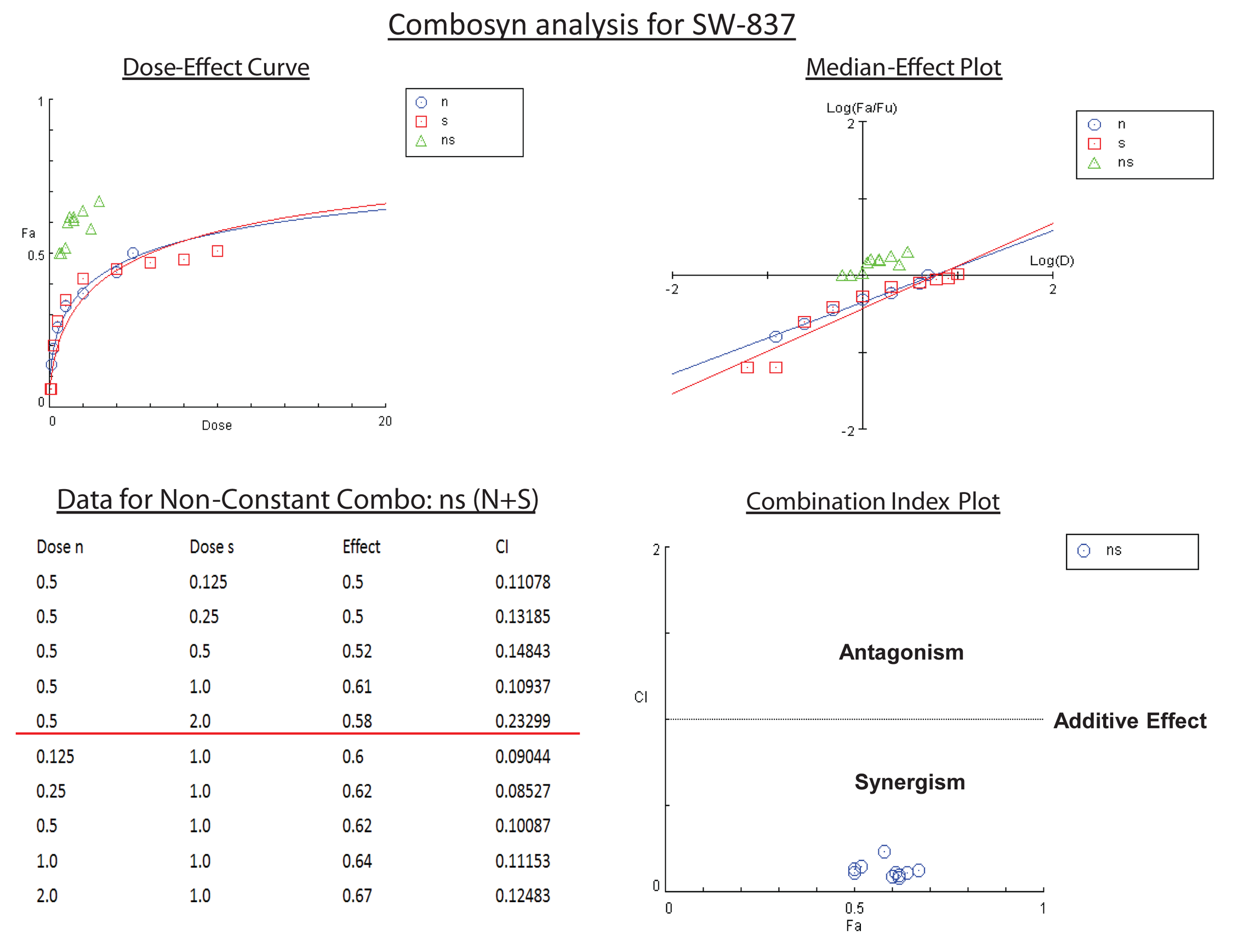

Supplement: S10 Fig — (TIF) [file pone.0200836.s010.tif]

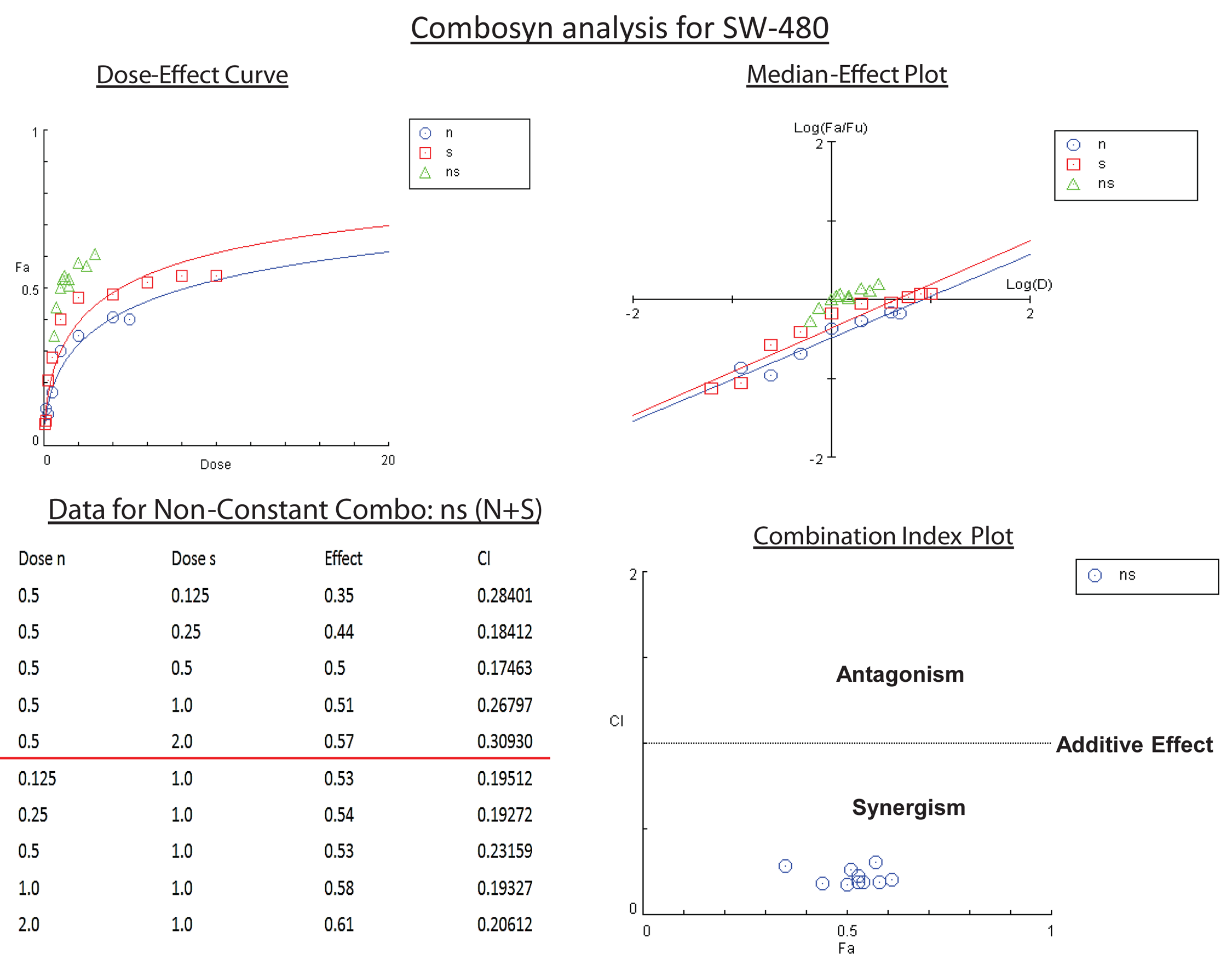

Supplement: S11 Fig — (TIF) [file pone.0200836.s011.tif]

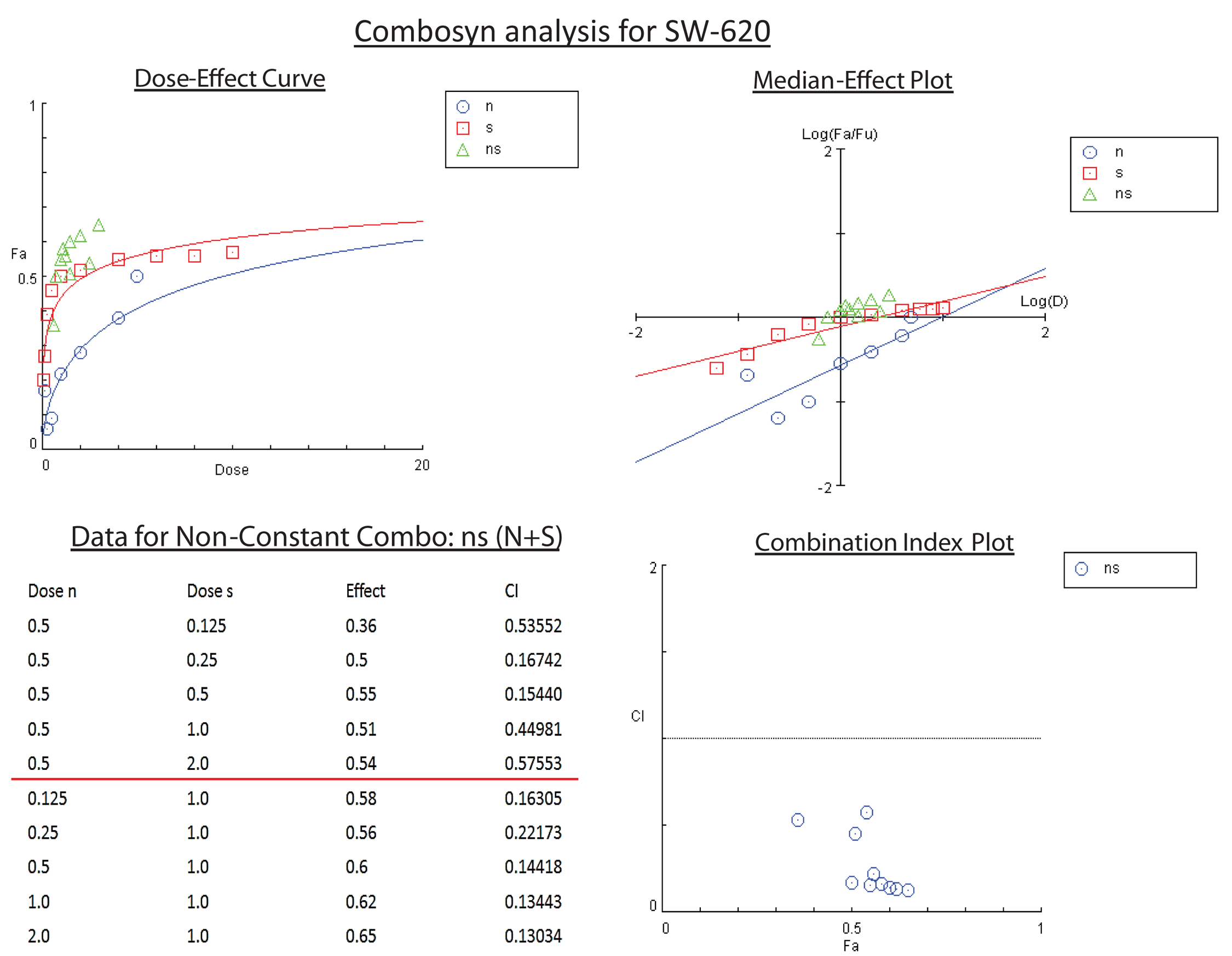

Supplement: S12 Fig — (TIF) [file pone.0200836.s012.tif]
