# Supplementary material for: Molecular subtypes of colorectal cancer in pre-clinical models show differential response to targeted therapies: Treatment implications beyond KRAS mutations
Source: PLoS One. 2018 Aug 17;13(8):e0200836. doi: 10.1371/journal.pone.0200836 (PMC6097647; doi:10.1371/journal.pone.0200836)
Supplement: S2 File — (PDF) [file pone.0200836.s014.pdf]

S2 File: Uncropped Western Blots / Raw Data for Figures 4 and 6

SFig 4A_B.1 p-ERBB2
SFig 4A-B.2 ERBB2
SFig 4A_B.3 p-ERBB3
SFig 4A_B.4 ERBB3
SFig 4A_B.5 p-ERK
SFig 4A_B.6 ERK
SFig 4A_B.7 p-EGFR
SFig 4A_B.8 p-90RSK
SFig 4A_B.9 p-AKT
SFig 4A_B.10 AKT
SFig 4A_B.11 B-actin
SFig 4C.1 p-ERBB2
SFig 4C.2 ERBB2
SFig 4C.3 p-ERBB3
SFig 4C.4 ERBB3
SFig 4C.5 p-ERK
SFig 4C.6 ERK
SFig 4C.7 p-EGFR
SFig 4C.8 p-90RSK
SFig 4C.9 p-AKT
SFig 4C.10 AKT
SFig 4C.11 B-actin
SFig 6A.1 p-ERK
SFig 6A.2 ERK
SFig 6A.3 B-actin
SFig 6C.1 p-ERK
SFig 6C.2 ERK
SFig 6C.3 B-actin

Figure 4A

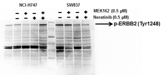

Figure 4B

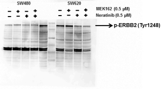

Figure 4A

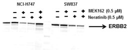

Figure 4B

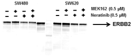

Figure 4A

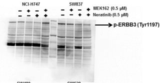

Figure 4B

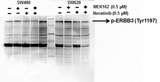

Figure 4A

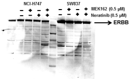

Figure 4B

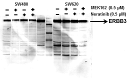

Figure 4A

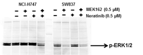

Figure 4B

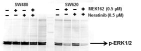

Figure 4A

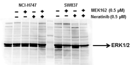

Figure 4B

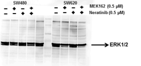

Figure 4A

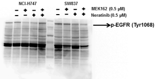

Figure 4B

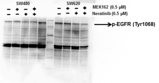

Figure 4A

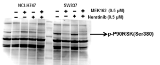

Figure 4B

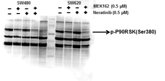

Figure 4A

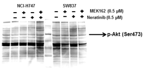

Figure 4B

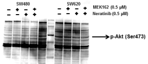

Figure 4A

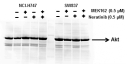

Figure 4B

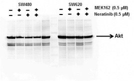

Figure 4A

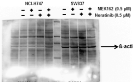

Figure 4B

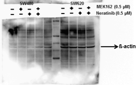

Figure 4C

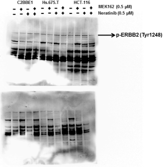

C2BBE1
Hs.675.T
HCT-116
MEK162 (0.5 µM)
Neratinib (0.5 µM)
p-ERBB2 (Tyr1248)

Figure 4C

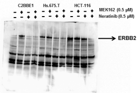

Figure 4C

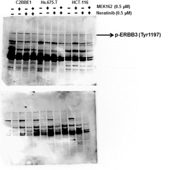

C2BBE1
Hs.675.T
HCT-116
MEK162 (0.5 μM)
Neratinib (0.5 μM)
p-ERBB3 (Tyr1197)

Figure 4C

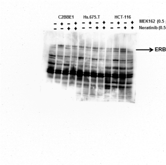

Figure 4C

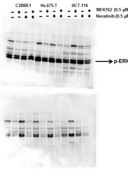

Figure 4C

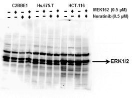

Figure 4C

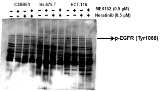
C2BBE1
Hs.675.T
HCT-116
MEK162 (0.5 μM)
Neratinib (0.5 μM)
p-EGFR (Tyr1068)

Figure 4C

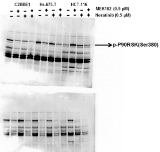
C2BBE1
Hs.675.T
HCT-116
MEK162 (0.5 μM)
Neratinib (0.5 μM)
p-P90RSK(Ser380)

Figure 4C

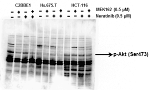
C2BBE1
Hs.675.T
HCT-116
MEK162 (0.5 μM)
Neratinib (0.5 μM)
p-Akt (Ser473)

Figure 4C

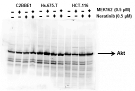

Figure 4C

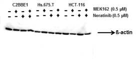

Figure 6A

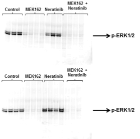
Control
MEK162
Neratinib
MEK162 + Neratinib
p-ERK1/2
Control
MEK162
Neratinib
MEK162 + Neratinib
p-ERK1/2

Figure 6A

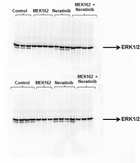

Control
MEK162
Neratinib
MEK162 + Neratinib
ERK1/2
Control
MEK162
Neratinib
MEK162 + Neratinib
ERK1/2

Figure 6A

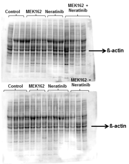

Control
MEK162
Neratinib
MEK162 +
Neratinib
ß-actin
Control
MEK162
Neratinib
MEK162 +
Neratinib
ß-actin

Figure 6C

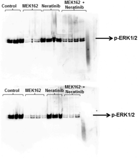

Control
MEK162
Neratinib
MEK162 + Neratinib
p-ERK1/2
Control
MEK162
Neratinib
MEK162 + Neratinib
p-ERK1/2

Figure 6C

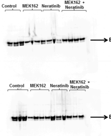
Control
MEK162
Neratinib
MEK162 + Neratinib
ERK1/2
Control
MEK162
Neratinib
MEK162 + Neratinib
ERK1/2

Figure 6C

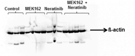

Control
MEK162
Neratinib
MEK162 +
Neratinib
ß-actin

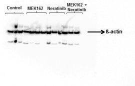

Control
MEK162
Neratinib
MEK162 +
Neratinib
ß-actin
